# The NASSS-CAT Tools for Understanding, Guiding, Monitoring, and Researching Technology Implementation Projects in Health and Social Care: Protocol for an Evaluation Study in Real-World Settings

**DOI:** 10.2196/16861

**Published:** 2020-05-13

**Authors:** Trisha Greenhalgh, Harvey Maylor, Sara Shaw, Joseph Wherton, Chrysanthi Papoutsi, Victoria Betton, Natalie Nelissen, Andreas Gremyr, Alexander Rushforth, Mona Koshkouei, John Taylor

**Affiliations:** 1 Department of Primary Care Health Sciences University of Oxford Oxford United Kingdom; 2 Said Business School University of Oxford Oxford United Kingdom; 3 mHabitat Leeds United Kingdom; 4 Department of Schizophrenia Spectrum Disorders (Psykiatri Psykos) Sahlgrenska University Hospital Mölndal Sweden

**Keywords:** evaluation, complexity, theory-driven evaluation, diffusion of innovation, scale-up, sustainability, implementation, NASSS (nonadoption, abandonment, scale-up, spread, sustainability) framework, innovation adoption, project management

## Abstract

**Background:**

Projects to implement health care and social care innovations involving technologies are typically ambitious and complex. Many projects fail. Greenhalgh et al’s nonadoption, abandonment, scale-up, spread, and sustainability (NASSS) framework was developed to analyze the varied outcomes of such projects.

**Objective:**

We sought to extend the NASSS framework to produce practical tools for understanding, guiding, monitoring, and researching technology projects in health care or social care settings.

**Methods:**

Building on NASSS and a complexity assessment tool (CAT), the NASSS-CAT tools were developed (in various formats) in seven co-design workshops involving 50 stakeholders (industry executives, technical designers, policymakers, managers, clinicians, and patients). Using action research, they were and are being tested prospectively on a sample of case studies selected for variety in conditions, technologies, settings, scope and scale, policy context, and project goals.

**Results:**

The co-design process resulted in four tools, available as free downloads. NASSS-CAT SHORT is a taster to introduce the instrument and gauge interest. NASSS-CAT LONG is intended to support reflection, due diligence, and preliminary planning. It maps complexity through stakeholder discussion across six domains, using free-text open questions (designed to generate a rich narrative and surface uncertainties and interdependencies) and a closed-question checklist; this version includes an action planning section. NASSS-CAT PROJECT is a 35-item instrument for monitoring how subjective complexity in a technology implementation project changes over time. NASSS-CAT INTERVIEW is a set of prompts for conducting semistructured research or evaluation interviews. Preliminary data from empirical case studies suggest that the NASSS-CAT tools can potentially identify, but cannot always help reconcile, contradictions and conflicts that block projects’ progress.

**Conclusions:**

The NASSS-CAT tools are a useful addition to existing implementation tools and frameworks. Further support of the implementation projects is ongoing. We are currently producing digital versions of the tools, and plan (subject to further funding) to establish an online community of practice for people interested in using and improving the tools, and hold workshops for building cross-project collaborations.

**International Registered Report Identifier (IRRID):**

DERR1-10.2196/16861

## Introduction

### Background

Technologies (which we define broadly as capabilities given by the practical application of knowledge) are often introduced in health care or social care settings as part of an attempt to improve services. Technology implementation projects (defined as active and planned efforts to mainstream a technology and associated changes to routines and services) have a high failure rate, especially when they are large, ambitious, and complex [1-4]. A previous study by our team explored the reasons why a very large, expensive, and centrally driven national program to implement an electronic patient record had failed to achieve its goals [5]. We concluded that such programs unfold as they do partly because nobody fully understands what is going on and that failure may result when this lack of understanding becomes mission-critical [6].

In that and other studies of large-scale innovation and technology implementation projects (see definitions), we have observed a tendency among policymakers and planners to employ bounded rationality—that is, to address an oversimplified and overly rationalized version of the challenge to make solutions seem more achievable [6-8]. Until recently, staff on many such projects had been trained in (and were expected to follow) the PRojects IN a Controlled Environment 2 approach [9], based on highly standardized procedures and a linear logic model with tightly stipulated goals and milestones. Significantly, we could find no health care or social care–based examples of such an approach in the academic literature. This is probably because the introduction of technology-supported change in health care and social care invariably involves not only technical implementation but also the ongoing judicious management of interacting subprojects characterized by competing values, goals, stakeholder interests, and local and national politics—all against a shifting contextual baseline [3,4,10-14].

To the extent that technology implementation is a rational and predictable process, both strategic planning and project evaluation can be target-focused and follow a logic model format (what we are trying to do, who will do it, by when, and so on) [15]. However, the introduction of new health care and social care technologies in real-world settings (with concomitant changes in organizational roles and routines) is more typically a social and political process in which power is unevenly distributed and success is defined differently by different stakeholders [15]. In such circumstances, relationships, interstakeholder negotiation, and collective sensemaking are crucial; contextual influences (both anticipated and unanticipated) cannot simply be *controlled for* or stripped out of the analysis [11,16-19].

*Complexity* has been defined as “a dynamic and constantly emerging set of processes and objects that not only interact with each other, but come to be defined by those interactions” [20]. *Health care innovation* has been defined as a set of behaviors, routines, and ways of working (along with associated technologies), which are perceived as new, linked to the management of a condition or the provision of services, discontinuous with previous practice (ie, not just quality improvement), directed at improving outcomes for service users and/or staff, and implemented by means of planned and coordinated action [20]. *Adoption* is the decision by an individual to engage with, and make full use of, an innovation. Innovations may spread and be implemented by *diffusion* (a passive phenomenon of social influence, leading to adoption), *dissemination* (active and planned efforts to persuade target groups to adopt an innovation), and *implementation* (active and planned efforts to mainstream an innovation) [20].

Complex systems have fuzzy boundaries; their interacting agents operate on the basis of internal rules that cannot always be predicted; and they adapt, interact, and co-evolve with other systems [21]. Complexity is a feature of the system, not merely a characteristic of interventions [22,23]. So-called complex interventions in health care (eg, the introduction of a patient-facing technology aimed at supporting evidence-based behavior change) and the context in which they are expected to have an impact (eg, a community with low health literacy and overstretched primary and secondary care services with high staff turnover) will inevitably be interrelated and reciprocally interacting.

**Figure 1:**
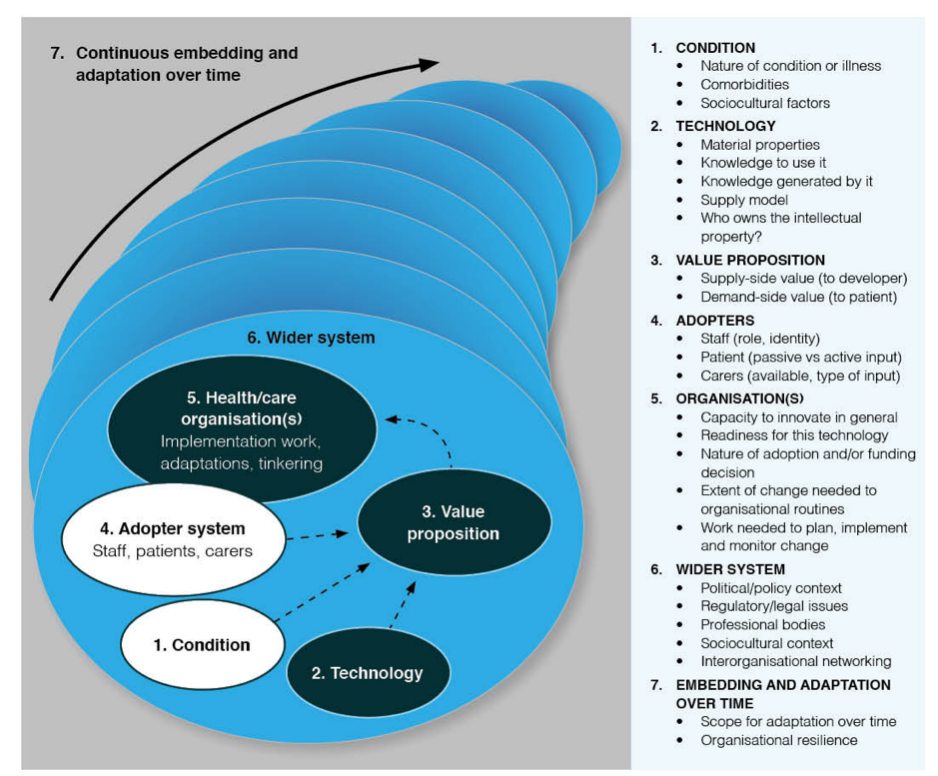
The nonadoption, abandonment, scale-up, spread, and sustainability framework for studying nonadoption and abandonment of technologies by individuals and the challenges to scale-up, spread, and sustainability of such technologies in health and care organizations.

We applied complexity principles in our work on the nonadoption, abandonment, and challenges to scale-up, spread, and sustainability (NASSS) framework whose theoretical development [24] and empirical testing [25] have been described in detail earlier. Briefly, we conducted a systematic literature review alongside six diverse case studies, which were explored longitudinally for 3 years using ethnography, interviews, and document analysis. The NASSS framework (see Figure 1) allows researchers to surface and explain the multiple forms and manifestations of complexity in technology-supported change projects. It consists of six domains—the condition or illness, the technology, the value proposition, the adopter system (intended users), the organization(s), and the wider system (especially regulatory, legal, and policy issues); the seventh, cross-cutting, domain considers how all these interact and emerge over time.

Although the NASSS framework has proved useful for illuminating and theorizing the successes, failures, and partial successes of technology implementation projects, it was designed for academic analysis, not as a practical tool for planning or managing technology projects prospectively. Such tools do, however, exist. Maylor et al [26], for example, developed a complexity assessment tool (CAT), on the basis that “[u]nderstanding and actively managing project complexity has the potential to identify better processes, staffing, and training practices, thereby reducing unnecessary costs, frustrations, and failures”.

In developing their original CAT, Maylor et al [26] viewed complexity as something that was subjectively perceived and experienced by managers (as opposed to an abstract property of the system—though it may be that too, and as something that evolves dynamically and more or less unpredictably over time. In addition to a systematic review on complexity in project management [27], Maylor et al [26,28] asked over 120 managers *What makes your project complex to manage?* recognizing that there would inevitably be multiple answers to this question. They distinguished three broad kinds of complexity:

- Structural—related to scale, scope, level of interdependence of people or tasks, and diversity of user requirements
- Sociopolitical—related to the project’s importance and its people, power, and politics (both within the project team and across wider stakeholders)
- Emergent—related to how stable the aforementioned issues are predicted to be over time

Maylor et al’s [26] original CAT consisted of 32 items, each a statement with which the respondent could agree or disagree. Of the 32 items, 21 related to structural complexity (eg, *The scope can be well-defined*) and 11 to sociopolitical complexity (eg, *Your own senior management supports the work*). Emergent complexity was assessed for each item by the additional question *Do you expect this situation to remain stable over time?*

Maylor et al’s [26] empirical work (undertaken across a wide range of companies outside the health care sector) showed that managers with limited domain knowledge typically did not experience a project as complex until their knowledge increased; managers on the same project often described the work quite differently (each identifying different elements of complexity but failing to recognize other elements); and they rarely considered a task to be complex unless or until they had some personal responsibility for delivering on it. Although project complexity might, in general, be expected to fall over time as unknowns become known and uncertainties shrink, in reality, projects often became *more* complex because of major changes in requirements, abandonment of work by delivery partners, and technical challenges with integration [26].

Maylor et al’s [26] rationale for producing the CAT was that if complexity could be better understood by project participants, it could often be reduced or actively managed. The CAT, which was field tested in 43 workshops involving over 1100 managers, was oriented toward a three-stage process—understand, reduce, and respond. These stages could be operationalized using CAT as a self-assessment and orientation tool, along with consultancy support, where needed [28]. The authors were surprised that in most cases, managers were able to identify strategies that allowed them to reduce the majority of complexities that they faced.

In sum, Greenhalgh et al’s [24] NASSS and Maylor et al’s [28] CAT, which were developed independently (one in health services research and one in business studies), for different purposes, and without knowledge of each other’s work, were both centrally concerned with exploring complexity (eg, identifying challenges, uncertainties, and interdependencies) in technology projects. Both tools included questions about operational logistics and about the human and political aspects of projects, and both included a cross-cutting domain to assess emergence over time.

### Objectives

In this new study, our aims are both methodological and empirical. Methodologically, we have sought to combine our programs of work to develop, validate, and extensively test a new instrument (NASSS-CAT) for understanding, reducing, and responding to complexity in the health (and health-related social care) sectors. Empirically, we are using NASSS-CAT to support, and generate lessons from, the implementation, routinization, spread, scale-up, and sustainability of technology-supported change in health care and social care. 

Our research questions were as follows:

1. Is it feasible and helpful to combine the NASSS framework with CAT that will help with understanding, guiding, monitoring, and researching technology implementation projects in health care and social care?
2. To what extent can the use of NASSS-CAT tools enable the multiple aspects of complexity in health care and social care technology projects to be identified, reduced, and actively managed by policymakers, planners, and project teams?
3. How might the NASSS-CAT tools be used in practice (eg, who should complete the tool, when and how, and is a trained facilitator needed)?

We answer the first of these questions in the following sections, based on work completed to date. In addition, we describe a protocol for answering the second and third questions in a new set of case studies that are currently ongoing.

## Methods

### Origins, Management, and Governance of the Study

The initial groundwork to develop the NASSS framework was undertaken as part of our SCALS (Studies in Co-creating Assisted Living Solutions, 2015-2020) and VOCAL (Virtual Online Consultations–Advantages and Limitations, 2015-2017) research programs, whose methodology [29,30] and main findings [24,25,31,32] have been reported earlier. Co-design work to develop NASSS-CAT, described in the next section, was undertaken as part of SCALS. Recruitment of six case studies for prospectively testing NASSS-CAT was undertaken as part of a wider program of translational research within the Oxford Biomedical Research Centre [33]. All these programs have (or had) external steering groups with a lay chair and a wide range of stakeholders from UK National Health Service (NHS), social care, industry, academia, and patients. Steering groups meet six-monthly and receive a three-monthly interim report.

The study received research ethics approval from the Health Research Authority and Health and Care Research Wales on June 21, 2019, and from the National Research Service Permissions Coordinating Centre for Scotland on August 9, 2019 (IRAS no. 258679; REC no. 19/LO/0550). Many but not all research ethics committees have deemed the use of the NASSS-CAT *service evaluation* rather than *research*. This reflects an inherent ambiguity in the project: the tools are indeed designed to support service implementation, but there are also research questions (set out above), which seek to generate generalizable findings relating to their use in service evaluation.

### Co-Design Phase: Developing and Refining the Nonadoption, Abandonment, Scale-Up, Spread, and Sustainability–Complexity Assessment Tools

This phase was undertaken in 2018-2019. TG and HM mapped Maylor et al’s [26] original CAT questions to the seven NASSS domains, merging duplicates from the two instruments and eliminating those irrelevant to health care or social care. This resulted in an early draft of the NASSS-CAT instrument.

A nonprofit digital consultancy firm, mHabitat, which specializes in improving success of public sector technology projects, held six 3-hour workshops involving 42 participants drawn from health care, social care, patient organizations, technology suppliers, and wider stakeholders. They were involved in digital projects, which spanned all stages, from idea to implementation. Workshop participants used design techniques (1) to share examples of health and care projects for which technological solutions were being or had been developed, (2) to analyze these examples by applying a set of structured questions from the draft NASSS-CAT instrument, (3) to reflect on whether and to what extent the questions had helped them identify the complexities in their projects, and (4) to provide feedback about usability and suggest improvements. Written notes, flip charts, and photographic records from each workshop were retained and summarized. A seventh workshop of 2 hours was held with a panel of 8 patients and carers interested in digital technologies in health care (see the Patient and Public Involvement section).

In response to multiple requests from PhD students and researchers, the NASSS-CAT tools were developed into a semistructured interview guide (NASSS-CAT INTERVIEW).

### Selection of Case Studies for Testing the Nonadoption, Abandonment, Scale-Up, Spread, and Sustainability–Complexity Assessment Tools

At the time we were developing the NASSS-CAT tools, we were approached (usually by email and also in lectures or workshops where we were presenting our work) by around 20 teams seeking to use the NASSS framework to support technology implementation. This offered us the potential to test and further refine the NASSS-CAT tools on real-time, real-world projects. We did not have the capacity to support all these projects, so we defined a smaller, purposive sample to provide maximum variety in the following criteria: target population, nature (and system implications) of the condition and the technology, sector (health and/or social care), policy context (policy *push* vs policy negative or neutral), and geographical setting (including non-UK examples). All case studies selected for the testing phase were characterized by a successful proof-of-concept pilot or demonstration project and a strategic decision to attempt local scale-up or distant rollout, both with a view to achieving long-term sustainability.

The cases whose characteristics are summarized in Multimedia Appendix 1 (also see the Summary of Case Studies for more details) illustrate a wide range of challenges in digital health care and social care.

A final selection criterion for our sample of case studies was relevance to our interest in intervention-generated inequalities (IGIs). These arise when only the more digitally capable and digitally equipped members of the target group gain full access to the technology’s benefits. As Veinot et al [34] have commented:

Many health informatics interventions may not themselves address social factors contributing to health disparities, such as poverty, residential segregation, and discrimination. However, they carry a risk of creating IGIs, and thus worsening underlying inequalities. We propose that such IGIs can be minimized or prevented through thoughtful decisions about access, uptake, adherence, and effectiveness.

As we are committed to redressing the tendency of technology research to contribute to IGIs, we deliberately selected several case studies that seek to reduce such inequalities, for example, by extending the use of video consultations to underserved groups or adapting video technologies for the elderly *analog generation*.

### Summary of Case Studies

#### Case Study 1: A Digital Dashboard to Increase Engagement in Evidence-Based Schizophrenia Care

This study is based in a secondary care psychiatry service in Gothenburg, Sweden. Schizophrenia is a serious and usually lifelong psychotic condition that typically begins in young adulthood. Treatment includes medication, psychological support, and social support, but compliance with all can be poor. A digital dashboard enabling visualization of key indicators of each patient’s health and care status (including structured questionnaires to help evaluate care at medical encounters) was developed with a view to encouraging more active involvement of patients in their own care. Despite a strong coproduction component, scale-up and deployment of the dashboard proved difficult.

#### Case Study 2: Technology to Support the European Union Falsified Medicines Directive

Falsified and counterfeit medicines are a hazard and occur in every country [35]. With the aim of protecting health and also ensuring sustainability of the European pharmaceutical market, changes to European Union (EU) law were introduced by the Falsified Medicines Directive 2011 (FMD 201162/EC) [36,37]. EU countries, along with the United Kingdom, are committed to implementing the directive. This means introducing, in every pharmacy, a technological solution to support two obligatory safety features: (1) a unique identifier for every package of medicine, and (2) an antitampering device. It also means up-front investment by every pharmacy in hardware and software that can verify the end-to-end supply chain from the manufacturer to the point of supply to the patient. Use of these technologies will require substantial changes to organizational routines and procedures. Preliminary data indicate that few community pharmacies in the United Kingdom are fully prepared for this change. Successful implementation is likely to be influenced by numerous factors, including the attitudes and capabilities of individual pharmacists and the changing structure of pharmacy provision in the United Kingdom (eg, the move from owner pharmacists to corporate chains).

#### Case Study 3: Video-Mediated Social Connection Technologies

Loneliness (a subjective feeling of lack of social contact) and social isolation (weak or absent social networks) are increasing in older people, many of whom live alone [38]. This is addressed as a government priority in the United Kingdom [39]. Communication with family via a video link can reduce both loneliness and social isolation [38]. A number of technologies have been designed for the *analog generation* (eg, they resemble old-fashioned TV or radio sets and have one or two large buttons). Some are already in use by private purchasers, but as yet, no public sector provider has invested significantly in them. We have begun to work with two suppliers, along with selected social care providers and care homes in both the United Kingdom and Norway, to explore how video technologies may be used more widely as part of a strategy to reduce loneliness.

#### Case Study 4: Digital Support for Cancer Multidisciplinary Team Meetings

Multidisciplinary team (MDT) meetings are generally considered the gold standard in cancer services, but as workloads have increased in recent years, they have become overcrowded and inefficient [40,41]. A third-party digital solution offers *end-to-end* support for the MDT meeting, including organizing data collection, visualizing materials (biopsies, scans, and blood test results) on-screen during the meeting, and inserting a summary and decision into the patient’s electronic record. Research suggests that efficiency and safety gains could be considerable [42,43], but again, the technology appears disruptive and real-world implementation has been little studied.

#### Case Study 5: Extending Video Consultations to Become Business as Usual

Our previous research demonstrated the potential for some outpatient and primary care consultations to be undertaken effectively and safely by a video link [31,32]. However, it also showed that such consultations are only offered to a small percentage of eligible patients (often excluding those with limited English, low health literacy, complex health and social needs, or no internet connection at home). We are now working with various public sector and third-sector providers in England, Scotland, Wales, and Australia to support efforts to increase access to video consultations for patients with a much wider range of clinical conditions and also for underserved and underresearched groups whose various needs raise a range of logistical, technical, ethical, cultural, and clinical challenges.

#### Case Study 6: Digitization of Histopathology Services

This case addresses the introduction, mainstreaming, and regional spread of whole-slide imaging and related technologies in histopathology. Glass slides (eg, surgical biopsies) are scanned into a computer and viewed on screen; they can be retrieved easily and transmitted electronically for specialist opinions. Research suggests that substantial improvements in service efficiency and safety can occur [44,45], and it is hoped the change will help address a serious and worsening workforce crisis in pathology [46]. Due to major implications for workflows and staff roles, digitization of histopathology services is seen as a *disruptive* innovation, which poses daunting challenges for departments.

### Early Piloting in Case Study 1

Earlier versions of the NASSS-CAT tools were used in case study 1 (conducted during 2018-2019 in Sweden; see Multimedia Appendix 1 and the Summary of Case Studies). Detailed methods and findings for that study have been published recently [47]. In short, the project goal was to develop a patient portal for people with psychosis. NASSS-CAT was used to structure a 1-day interprofessional workshop attended by 11 participants (line managers, department directors, organization developers, programmers, information technology developers, and clinical professions such as psychiatrists, psychologists, and occupational therapists). The day included both small breakout group sessions and large-group discussions. Outputs included free text mapping of different aspects of project complexity onto the NASSS domains. These were subsequently presented at two feedback sessions to senior and frontline staff involved in planning the future deployment of the technology and used to guide next-step planning.

### Action Research in Case Studies 2 to 6

We are currently undertaking groundwork with five other project teams for a set of in-depth, longitudinal case studies in the United Kingdom using the principles of action research [48,49]. This comprises gaining access to the cases, building relationships and mutual understanding (especially of how the different versions of NASSS-CAT can be used to plan, guide, monitor, and evaluate the project), and agreeing on potential data sources and collection methods for monitoring process and outcomes (including data to populate costing models). The main method used at this stage is informal interviews and visits along with attendance at routine meetings; more formal audiotaped interviews will be undertaken where appropriate.

In the next phase, with different case studies commencing at different times over the next 3 to 12 months, we will work collaboratively with health care and social care teams and technology suppliers to support and monitor the implementation of the technological innovation and efforts to achieve sustained changes in work routines and system processes. This will include regular meetings for providing feedback on interim findings, responding to unforeseen events, and capturing learning. In this main action research phase, we will periodically administer the NASSS-CAT PROJECT instrument (see next sections for details) to a sample of project managers and other stakeholders and, following the approach developed by Maylor et al [26,28], generate quantitative data on how the perceived complexity of each project changes over time.

For each case, we plan to collect high-quality data to inform a quantitative before-and-after comparison (with and without the new technology). Metrics will be different in each case and iteratively adapted and will be described in detail in separate, detailed publications for each case. Quantitative data sources may include, for example, usage statistics, waiting times, and proportion of a defined denominator population who are confident users of the technology. All case studies have received some initial funding (see the Acknowledgments section). In some studies, continuation of data collection and analysis for the full study period will depend on securing additional research funding. We plan to follow these five case studies from 2019 to a planned completion date of 2022.

### Stakeholder Interviews

We are also undertaking a wider (national and international) case study of the context for innovation. Building on our existing contacts, we will gain access to policymakers (including NHS England, NHS X, NHS Digital, and Health Education England), industries (both large and small technology companies), professional bodies (eg, Royal Colleges and General Medical Council), and patient and advocacy organizations. We will conduct background stakeholder interviews and maintain a two-way dialogue with these stakeholders throughout the study and link them with our dissemination efforts.

### Analyzing and Theorizing

Principally, through the various theoretical perspectives that have been built into the NASSS-CAT instrument, we will apply relevant theories of technology adoption, implementation, spread and scale-up, and specific change and monitoring tools. These include theories of individual behavior change (to explain nonadoption and abandonment by individuals), organizational capacity to innovate and readiness for change, technologies as part of complex systems, and value creation. Using empirical data and cross-case synthesis from the case studies, we will refine and extend the NASSS-CAT tools and linked resources, embracing additional approaches where appropriate.

### Patient and Public Involvement

We are committed to patient and public involvement in all stages of this research. We have recently established a standing panel, Patients Active In Research on Digital health (PAIReD) with diversity in age, ethnicity, gender, and educational background. A member of PAIReD (JT) is a coauthor of this protocol. The action research process in each case study (still to be defined in detail) will include contextually appropriate methods for gaining input from patients and service users, including comments on data sources and input to data analysis and action planning. The PAIReD panel, and our wider online network of Patients Active In Research, will be consulted on dissemination activity, especially the preparation of lay summaries and a public-facing website.

## Results

### Co-Design Phase to Refine the Nonadoption, Abandonment, Scale-Up, Spread, and Sustainability–Complexity Assessment Tools

The co-design workshops generated a great deal of data that allowed us to refine the individual questions on NASSS-CAT. Most participants reported that they found the tools useful and felt that they had gained valuable insights into their technology project (or idea for a project) by using it. Specific issues raised that informed iterative refinement of the tools between workshops included:

- Rationale: Participants suggested an introductory section that explained what the tool was, how it had been developed, and how it was intended to be used.
- Terminology: Participants from different backgrounds were confused by terms used in different domains. For example, some people with a technical background had limited understanding of clinical terms, and many clinicians did not understand questions about business models. Not everyone knew what intellectual property was or what a value chain meant.
- Readability: The workshops identified long sentences, double negatives, and jargon, which were removed in subsequent iterations.
- Length: Adding the various explanations proposed by some participants made the tool very long; therefore, a later group proposed developing an additional short, *taster* version.
- Scoring: Earlier versions had a binary (yes or no) scoring system, but often, the response was *it depends* or *to some extent;* therefore, intermediate options were created.

Some of the co-design feedback was difficult or impossible to incorporate into paper (or PDF) versions of the tools, but we plan to address this issue in a future digital version which is now in production. For example:

- Order of questions: Stakeholders held different views about which order the domains should be listed in. A digital version could have multiple entry points designed for different users.
- Expandable format: The long version of the tool was off-putting and contained long sections that were irrelevant to some projects (eg, some technologies are not condition specific, so domain 1 is redundant). But the short version was too brief for a meaningful analysis of a real-world project. A digital version could be short but have hypertext links to be pursued if relevant.
- Use cases: The co-design workshops generated much discussion on *how* (ie, by whom) and *when* (ie, at what stage in the project) the NASSS-CAT tools might be used. People whose projects were advanced felt the version evaluated would have been useful at an earlier stage, as they felt it summed up the experience they had already gained. Those who had not yet begun felt what many of the questions were premature. A digital version of the tool could identify the phase of the project and take the user to an appropriate set of questions.
- Automatic score tally: Some participants did not want to add up the scores. A digital version could do this automatically and in real time.
- Visualization: Participants suggested various graphics, including histograms and radar charts, which could be incorporated into a digital version.
- Additional resources: The co-design workshops surfaced numerous existing resources, which (if included) would make a paper or PDF version of the tool unwieldy but to which a digital version could link. These include Web-based project management guides and templates, a digital assessment questionnaire designed to guide due diligence and risk assessment before investing in a technology, sources of information about specific diseases or conditions, regulatory standards, and co-design tools for incorporating the patient experience into the design of technologies and care pathways.

Four versions of NASSS-CAT have been produced to date as follows:

- NASSS-CAT SHORT (reproduced in Multimedia Appendix 2), a 3-page taster instrument, in paper (or PDF) format, designed to introduce the instrument and gauge interest. It is semiquantitative in that it seeks *agree/disagree a little/disagree a lot* responses on a range of questions relating to the different NASSS domains.
- NASSS-CAT LONG (Multimedia Appendix 3), a more detailed version of the tool, in paper/PDF and Web-based format (the last is still under development). This version is intended to be used at the stage when there is an idea, a suggestion, or a broad goal to introduce a technology, but there is no formal, agreed project yet. NASSS-CAT LONG can be used for detailed reflection and preliminary project planning, usually (though not necessarily) with support from a trained facilitator. It invites discussion among the project’s many stakeholders across six domains, using interpretive (free-text) questions designed to generate a rich narrative and surface uncertainties and interdependencies and survey (closed-item) questions for systematically assessing different kinds of complexity. There is also an action planning component aimed at shaping the early ideas into a formal project, including due diligence (eg, assessment of quality, safety, and regulatory issues) for the technology.
- NASSS-CAT PROJECT (Multimedia Appendix 4) is a 35-item instrument for monitoring the complexity of a technology implementation project (as perceived by project team members) over time. Five kinds of project-related complexity are considered: strategic, technical, operational, people related (eg, human resources), and political. Project teams may use this tool in a variety of ways—perhaps in an initial in-depth kick-off workshop followed by periodic reviews—and usually with a trained facilitator or project consultant.
- NASSS-CAT INTERVIEW (Multimedia Appendix 5) is a set of prompts for conducting semistructured interviews (eg, by someone doing research into the implementation of a technology).

### Case Study 1: Patient Portal for People With Schizophrenia

In case study 1, the NASSS-CAT workshop generated rich data that informed the early stages of the project. Complexity mapping revealed, for example, that while intended adopters (staff and patients) were engaged and keen, there were high levels of complexity in all other domains, including the illness (schizophrenia is a heterogeneous condition with unpredictable course and often multimorbidity and associated social problems; the portal has significant interdependencies with systems controlled by third parties; the value proposition for the technology was uncertain; while departmental tension for change was high, the dashboard did not appear to be a strategic priority for the organization as a whole and the business plan was not considered persuasive; despite a strong pro-technology policy push in Swedish health care, the number of new products competing for attention may have overshadowed the portal project; and the practicalities of implementation appeared complex).

Although the mapping exercise did not generate easy fixes, workshop participants found that surfacing and talking through the complexities were extremely useful for clarifying and working through the project at a time when progress was slow. Recommendations stemming directly from the NASSS-CAT workshop in this case included (1) developing a clear value proposition with information on costs, benefits, and risks; (2) developing and disseminating a rolling shared vision of what the project is and keeping this updated; (3) strengthening project leadership and governance and allocating a dedicated budget to it; (4) focusing initially on the less complex components and functions of the technology; and (5) acting strategically in the wider context (eg, by seeking to rebrand the project to fit a policy initiative). Suggestions for improving the process included attention to detail in advance of the workshop to define each term in the NASSS-CAT tool more precisely in relation to the specific project being discussed. The participants also suggested including additional staff groups in the workshop.

Results of the other five (ongoing) case studies will be presented in subsequent publications.

## Discussion

This protocol has described how we combined a theory-informed analytic framework to deepen understanding (NASSS) with a pragmatically focused planning tool to aid implementation (CAT) to produce the four versions of the NASSS-CAT tools and outlined the characteristics and data collection plans for a series of real-world case studies to test these tools. Our approach is based on the principle that if a project is complex, it is unlikely to be effectively managed using a linear, logic model methodology and technocratic progress metrics. On the contrary, the greater the uncertainties and interdependencies in a project, the more crucial it is to avoid oversimplifying and overrationalizing.

A limitation of the NASSS-CAT tools, according to those who favor a more rationalistic approach, is that they are relatively unstructured and likely to generate *messy* data. However, the strength of these tools, we believe, is that for the very reason that they are unstructured, they are particularly suited to addressing the hypercomplexity of many health and care technology projects.

In a recent theoretical paper entitled “Don’t Simplify, Complexify,” Tsoukas [50] warns against the temptation to produce a simplified and abstracted version of the challenge (an approach he calls *disjunctive theorizing*) and instead seeks to build a rich picture of the case in all its complexity by drawing together different kinds of data from multiple sources using a technique he calls *conjunctive theorizing*. Such an approach assumes an open-world ontology (ie, it sees the world as subject to multiple interacting influences, which must be captured in a rich and dynamic way), a performative epistemology (ie, it focuses on real-world action and on what becomes possible through action), and a poetic praxeology (ie, when writing up case studies, it seeks to produce descriptive details, an apt metaphor, and a narrative coherence) [50].

Our work to date on the NASSS-CAT tools has sought to embrace these features of conjunctive theorizing. The NASSS-CAT LONG, in particular, is designed to capture, through free-text narrative, the numerous interacting influences that could affect project success; to highlight the twists and turns as the project unfolds; and to foreground mundane issues that help explain why the project has stalled. The tool’s action-planning section is directed at Tsoukas’s [50] performative component (*what becomes possible through action*). Similarly, the NASSS-CAT INTERVIEW is designed to help a participant construct a sensemaking narrative of the (perhaps meandering) fortunes of a complex implementation project.

Another potential limitation of the NASSS-CAT tools is that people who do not understand the unresolvable nature of problems in complex systems may apply them in a rigid and deterministic way rather than—as we intend them to be used—flexibly and imaginatively to accommodate the wide differences between projects. In addition, the tools presented as appendices to this paper might be viewed as definitive rather than preliminary. We anticipate that as experience in using the NASSS-CAT tools accumulates, further refinements will be made. At this stage, we strongly encourage implementation teams and implementation researchers to view these as *beta versions* and provide us feedback in the form of suggestions for improvements in their design or application.

In the dissemination phase of this study, we aim to produce a range of written and other outputs for academic and lay audiences, including standard academic outputs (peer-reviewed journal articles, conference presentations) plain-language versions of more definitive NASSS-CAT tools, resources (such as customizable templates and facilitator guides), and policy briefings.

In conclusion, we have presented the first iteration of a suite of tools designed to apply complexity principles to understand, guide, monitor, and evaluate technology projects in health and care. Academic and service teams are already using these tools to help achieve implementation, spread, and scale-up of various kinds of technology-supported change. We hope to report both empirical and theoretical findings from the case studies described here and additional cases as these come onstream. We also encourage teams working in low- and middle-income countries to use these tools in formal research or evaluation work, to extend their current scope of application.

Interested colleagues are also asked to note that (subject to further funding) we plan to establish, support, and nurture a community of practice that welcomes both academic, practitioner/policy, and lay members who share our interest in applying, improving, and learning from the NASSS-CAT tools. We are, in principle, interested in providing support to other research groups and implementation teams who seek to use NASSS-CAT in technology implementation, although, in practice, this will depend on our availability (and probably on securing additional external funding).

## Appendix

Multimedia Appendix 1 Characteristics of the six case studies.

Multimedia Appendix 2 Nonadoption, abandonment, scale-up, spread, sustainability short.

Multimedia Appendix 3 Nonadoption, abandonment, scale-up, spread, and sustainability–complexity assessment tools long.

Multimedia Appendix 4 Nonadoption, abandonment, scale-up, spread, and sustainability–complexity assessment tools project.

Multimedia Appendix 5 Nonadoption, abandonment, scale-up, spread, and sustainability–complexity assessment tools interview.

## Abbreviations

CAT: complexity assessment tool
EU: European Union
IGI: intervention-generated inequalities
MDT: multidisciplinary team
NASSS: nonadoption, abandonment, scale-up, spread, sustainability
NHS: National Health Service
PAIReD: Patients Active In Research on Digital health
SCALS: Studies in Co-Creating Assisted Living Solutions
VOCAL: Virtual Online Consultations–Advantages and Limitations
