# Supplementary material for: The NASSS-CAT Tools for Understanding, Guiding, Monitoring, and Researching Technology Implementation Projects in Health and Social Care: Protocol for an Evaluation Study in Real-World Settings
Source: JMIR Res Protoc. 2020 May 13;9(5):e16861. doi: 10.2196/16861 (PMC7254278; doi:10.2196/16861)
Supplement: Multimedia Appendix 1 [file resprot_v9i5e16861_app1.pdf]

TABLE 1: CHARACTERISTICS OF THE SIX CASE STUDIES						
**Criterion**	**Case 1: Patient portal for people with schizophrenia**	**Case 2: Digitisation of multidisciplinary cancer meetings**	**Case 3: Extending video consultations to underserved groups**	**Case 4: Support for EU Falsified Medicines Directive**	**Case 5: Video-mediated social connection technologies**	**Case 6: Digitisation of histopathology services**
Country / geographical setting	Sweden	UK (South East)	UK (inner London, Scottish Highlands, south Wales) and remote parts of Australia	UK-wide	Various localities in UK and Norway	UK (South East)
Target population and condition	Adults with psychosis registered with a specialist public mental health service	Patients with known or strongly suspected cancer	Outpatients who have rarely been offered video consultations in the past e.g. limited English speakers, prisoners, care home residents, abused women	All patients taking prescription medicines	Older people living alone who wish to have more social contact, especially with their relatives	Patients having surgical biopsies e.g. for suspected cancer
Technology	Point-of-care dashboard offering a visual overview of progress (including questionnaires for symptoms and functioning) and planning.	A third-party software tool that pulls data from the electronic record and allows visualisation of key cancer markers and images, thereby supporting team discussion	Video mediated communication software e.g. Skype™, Facetime™, AttendAnywhere™, used by staff on NHS desktops and by patients on their home computer or smartphone	Hardware and software to support end-to-end quality assurance of prescription medicines from manufacturer to patient supply in pharmacies.	For the older person, a ‘retro’ designed box that looks like a TV set (or which links to their own TV) and has a simple on-off and volume switch. For the relatives, a smartphone app	Whole-slide imaging and related technologies that allow digital storage, indexing and exchange of biopsy specimens
Sector	Secondary care	Secondary and tertiary care	Primary and secondary care NHS providers, prisons, care homes, third-sector settings	Hospital and community pharmacies	Social care	Secondary and tertiary care
Main goal of the project	Improving patient engagement with their own care; care planning; audit	Improving quality and efficiency of MDT meetings and ensuring timely recording of decisions in patient’s record	Extension of video consultation services to various underserved groups	Assuring the quality of medicines and building confidence in EU pharmaceutical suppliers	Uptake and roll-out of social connection technologies in different settings (e.g. care homes, private dwellings)	A fully digitised regional histopathology service; better use of specialist staff time
Policy context	High numbers of patients with schizophrenia, who are seen as a ‘vulnerable group’, with low engagement and compliance	Service under extreme pressure; rising case load	‘Care closer to home’ policy – video tech seen as potentially reducing outpatient load	EU Falsified Medicines Directive	Emerging national crisis of isolated and lonely older people	National workload and workforce crisis (shortage of pathologists)
Project framed primarily as	Empowering patients	Efficiency and safety	Increasing access to digitally-enabled services	Quality assurance and safety	Responding to demographic changes	Quality and efficiency
