# Supplementary material for: The NASSS-CAT Tools for Understanding, Guiding, Monitoring, and Researching Technology Implementation Projects in Health and Social Care: Protocol for an Evaluation Study in Real-World Settings
Source: JMIR Res Protoc. 2020 May 13;9(5):e16861. doi: 10.2196/16861 (PMC7254278; doi:10.2196/16861)
Supplement: Multimedia Appendix 2 [file resprot_v9i5e16861_app2.docx]

**NASSS-CAT (SHORT)**

**IDENTIFYING COMPLEXITIES IN YOUR TECHNOLOGY PROJECT**

The questions below help you think about the various complexities of your project and how they all interact. Use your responses and notes as the basis for a team discussion.

Name of your project: ……………………………………………………………………………………………………………………….

## THE ILLNESS OR CONDITION

*Think about the illness or other condition that the technology is designed for – and what sort of person has that condition.*

| 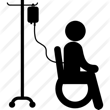 | *Agree* |  | *Disagree* | *Not applicable*  *or don’t know* | |
| --- | --- | --- | --- | --- | --- |
| There are significant uncertainties about the condition e.g. poorly-defined, variable manifestations, uncertain course |  | |  |  |  |
| Many people with the condition have other co-existing illnesses or impairments that could affect their ability to benefit from this solution |  | |  |  |  |
| Many people with the condition have social or cultural factors that could affect their ability to benefit from the technology or service |  | |  |  |  |
| The population with the condition, and/or how the condition is treated, is likely to change significantly over the next 3-5 years |  | |  |  |  |
| SUMMARY: The condition has significant complexity which is likely to affect the project’s success | *Yes* | | *No* | |  |

## THE TECHNOLOGY


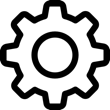
*Think about the technology (e.g. a tool or piece of software), and how it might affect care.*

|  | *Agree* |  | *Disagree* | *Not applicable*  *or don’t know* | |
| --- | --- | --- | --- | --- | --- |
| There are significant uncertainties in what the technology is (e.g. it hasn’t been fully developed yet) |  | |  |  |  |
| There are significant uncertainties in where the technology will come from (e.g. supply chain issues, substitutability) |  | |  |  |  |
| There are significant uncertainties about the technology’s performance and dependability (e.g. bugs, crashing, cutting out) |  | |  |  |  |
| There are significant uncertainties about the technology’s usability and acceptability (e.g. key people don’t trust the data it provides) |  | |  |  |  |
| There are significant technical interdependencies |  | |  |  |  |
| The technology is likely to require major changes to organisational tasks and routines |  | |  |  |  |
| The technology (and/or the service model it supports) is likely to change significantly within the next 3-5 years |  | |  |  |  |
| SUMMARY: The technology has significant complexity which is likely to affect the project’s success | *Yes* | | *No* | |  |

##
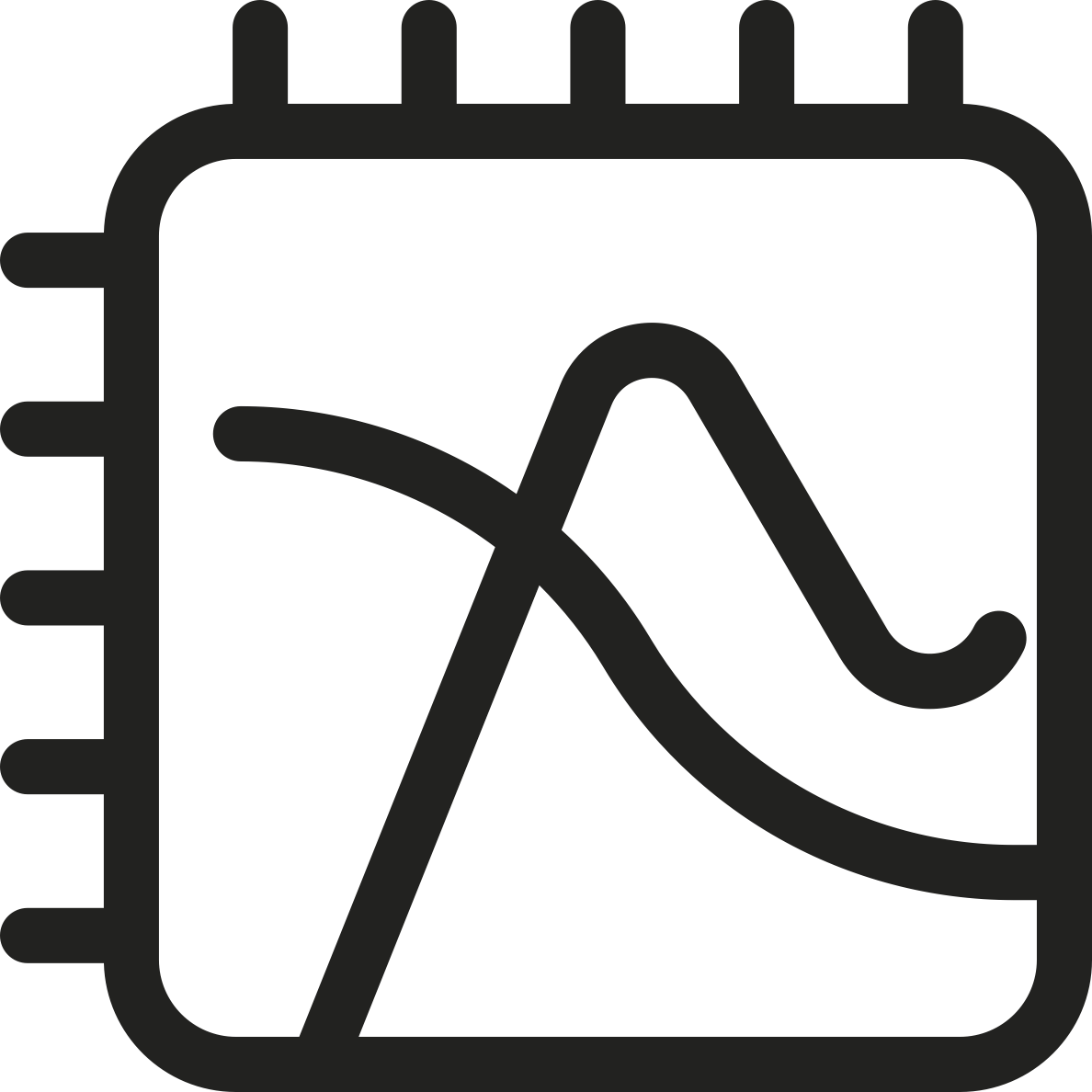
THE VALUE PROPOSITION

*Think about what kind of value the technology might generate for different groups of people. (‘Value’ can be financial, such as profit, or non-financial, such as control of symptoms)*

|  | *Agree* |  | *Disagree* | *Not applicable*  *or don’t know* | |
| --- | --- | --- | --- | --- | --- |
| The commercial value of the technology is uncertain |  | |  |  |  |
| The value to the intended users (e.g. patients, clinicians) is uncertain |  | |  |  |  |
| The value to the healthcare system (e.g. from efficacy and cost-effectiveness studies) is uncertain |  | |  |  |  |
| The value to this particular healthcare organisation, given the current situation locally, is uncertain |  | |  |  |  |
| The technology could generate a negative value (costs are likely to outweigh benefits) for some stakeholders |  | |  |  |  |
| The value proposition is likely to change significantly over the next 3-5 years |  | |  |  |  |
| SUMMARY: The value proposition has significant complexity which is likely to affect the project’s success | *Yes* | | *No* | |  |

##
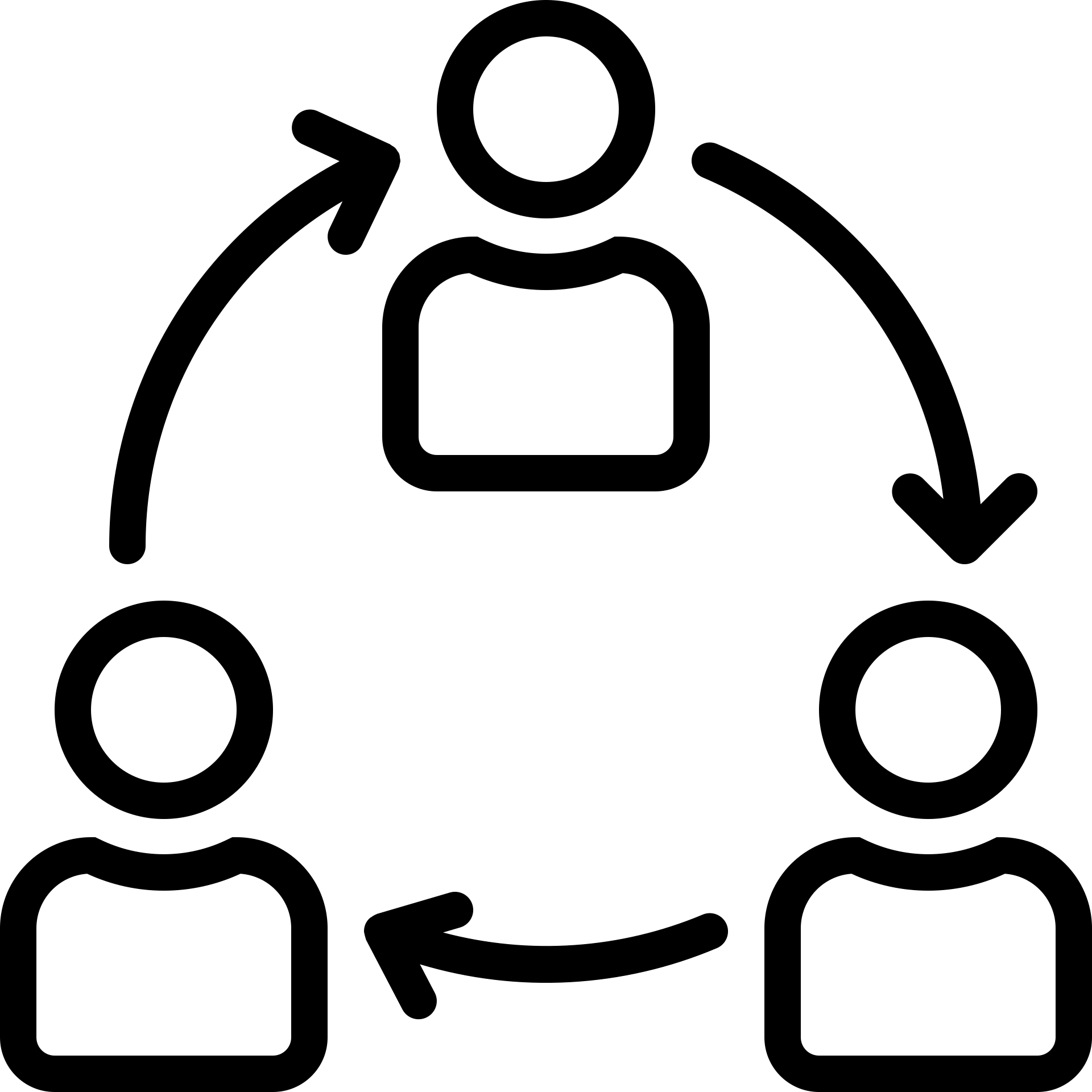
THE INTENDED ADOPTERS

*Think about who is intended to use the technology and what changes it will bring for them.*

|  | *Agree* |  | *Disagree* | *Not applicable*  *or don’t know* | |
| --- | --- | --- | --- | --- | --- |
| There is uncertainty about whether and how patients/citizens will adopt the technology [if applicable] |  | |  |  |  |
| There is uncertainty about whether and how front-line staff will adopt the technology |  | |  |  |  |
| There is uncertainty about the implications for people who might be indirectly affected by the technology |  | |  |  |  |
| There will be significant changes to individual users’ perceptions of the technology over the next 3-5 years |  | |  |  |  |
| SUMMARY: There is significant complexity relating to intended adopters which is likely to affect the project’s success | *Yes* | | *No* | |  |

##
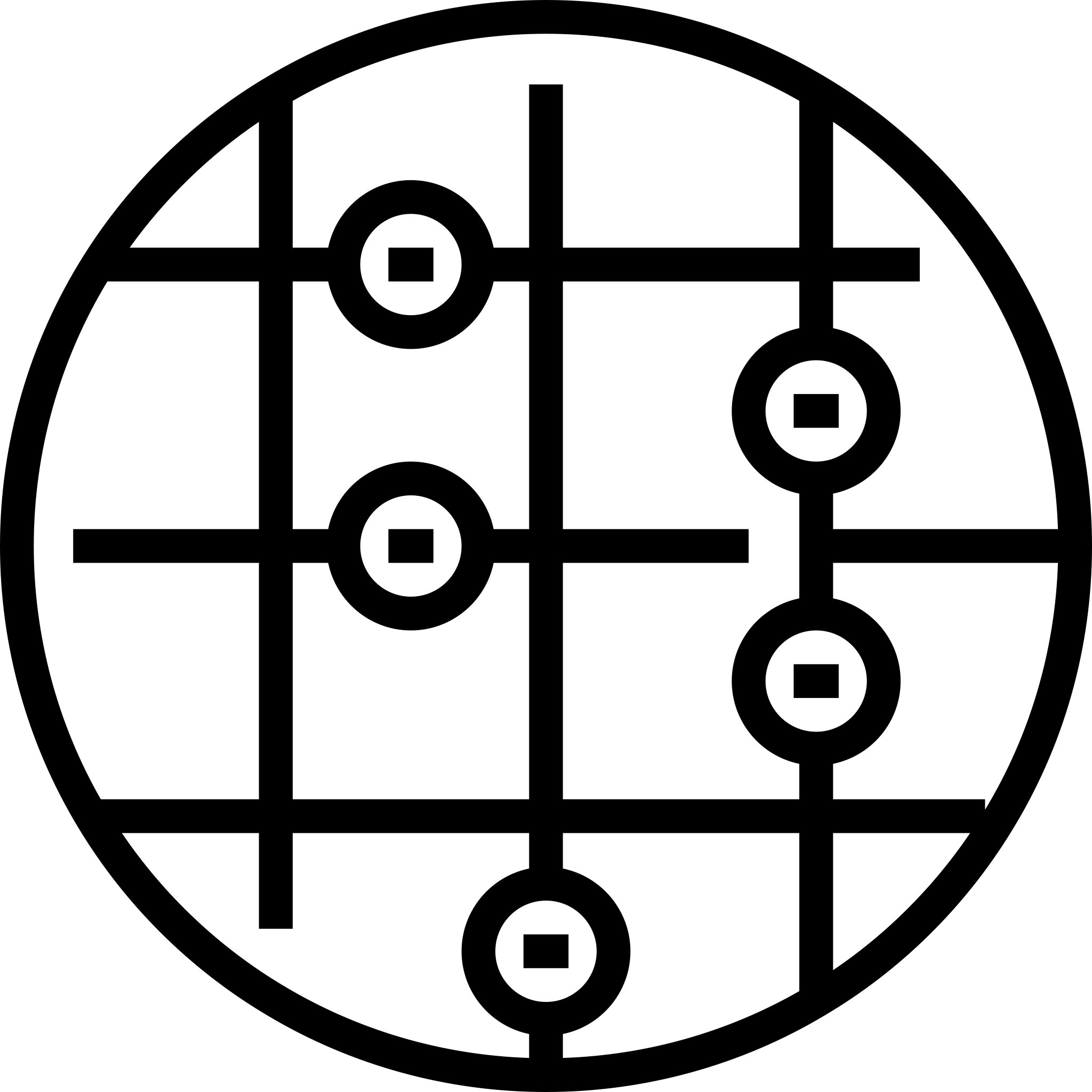
THE ORGANISATION(S) IMPLEMENTING THE TECHNOLOGY

*Some organisations are better at taking up innovations than others. What about yours?*

|  | *Agree* |  | *Disagree* | *Not applicable*  *or don’t know* | |
| --- | --- | --- | --- | --- | --- |
| The organisation’s capacity to take on technological innovations is limited |  | |  |  |  |
| The organisation is not ready for this particular innovation |  | |  |  |  |
| The organisation would find it hard to commission/purchase the innovation |  | |  |  |  |
| The work needed to introduce and routinise the innovation has been underestimated and/or inadequately resourced |  | |  |  |  |
| The organisation(s) involved are likely to have significant restructurings or changes in leadership, mission or strategy over the next 3-5 years |  | |  |  |  |
| SUMMARY: There is significant complexity relating to one or more participating organisations which is likely to affect the project’s success | *Yes* | | *No* | |  |

##
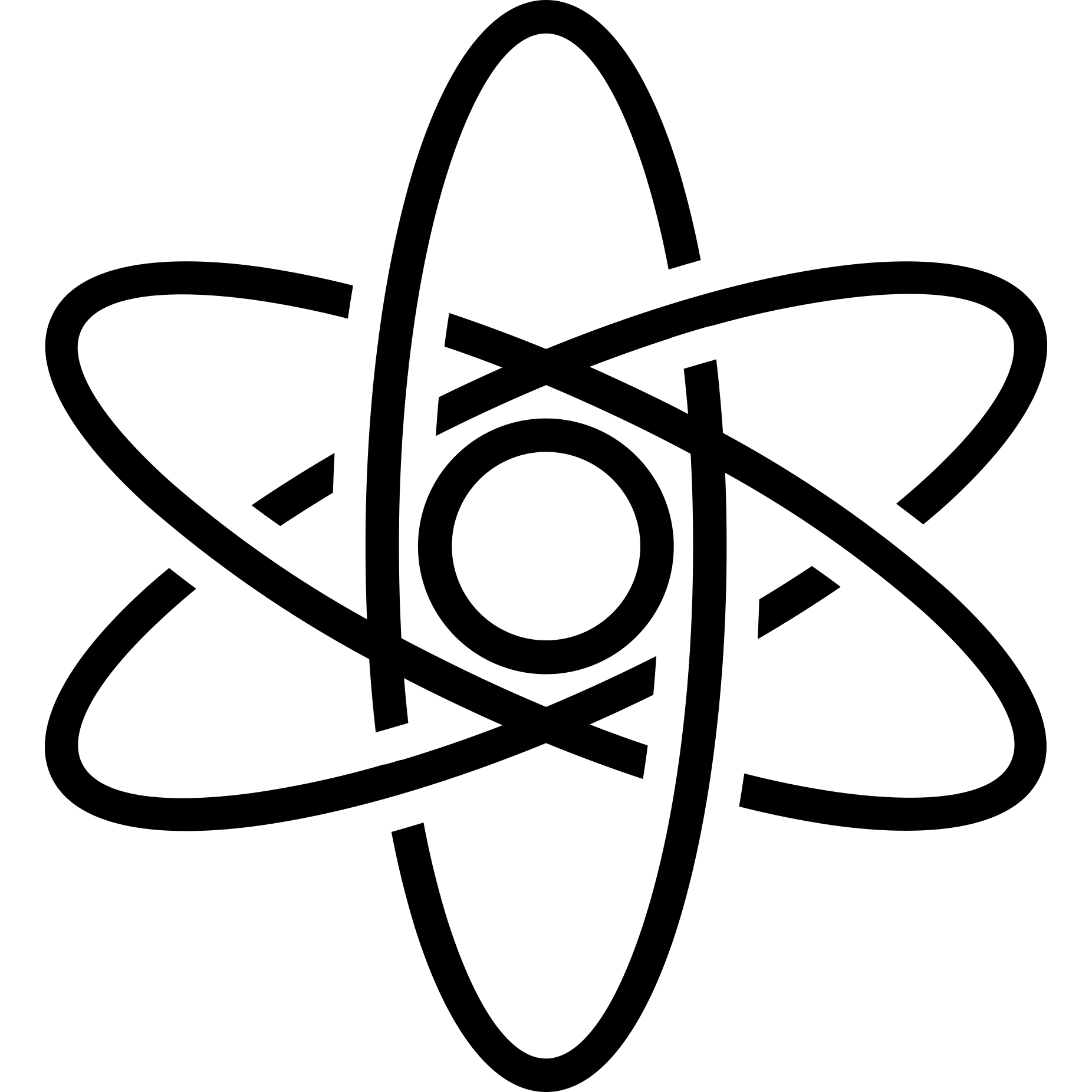
THE EXTERNAL CONTEXT FOR INNOVATION

*Think about external conditions that could complicate adoption and spread of the innovation.*

|  | *Agree* |  | *Disagree* | *Not applicable*  *or don’t know* | |
| --- | --- | --- | --- | --- | --- |
| The political and/or policy climate is adverse |  | |  |  |  |
| Professional bodies are opposed to the innovation or don’t actively support it |  | |  |  |  |
| Patient organisations and lobbying groups are opposed to the innovation or don’t actively support it |  | |  |  |  |
| The regulatory context is adverse |  | |  |  |  |
| The commercial context is adverse |  | |  |  |  |
| Opportunities for learning from other (similar) organisations are limited |  | |  |  |  |
| Introduction of the technology/innovation could be threatened by external changes that impact on the organisation |  | |  |  |  |
| The policy, regulatory and economic context for this innovation is likely to be turbulent over the next 3-5 years |  | |  |  |  |
| SUMMARY: There is significant complexity relating to the external context which is likely to affect the project’s success | *Yes* | | *No* | |  |

**THINGS TO EXPLORE OR DISCUSS: List the key things in each domain that you would like to look up or discuss with other team members or wider stakeholders**

The value proposition

The technology

The illness or condition

The organisation

The intended adopters

The external context
