# Supplementary material for: The NASSS-CAT Tools for Understanding, Guiding, Monitoring, and Researching Technology Implementation Projects in Health and Social Care: Protocol for an Evaluation Study in Real-World Settings
Source: JMIR Res Protoc. 2020 May 13;9(5):e16861. doi: 10.2196/16861 (PMC7254278; doi:10.2196/16861)
Supplement: Multimedia Appendix 3 [file resprot_v9i5e16861_app3.docx]

**NASSS-CAT (LONG VERSION)**

**Assessing and handling complexity in technology proJECTs**

© Professor Trish Greenhalgh, University of Oxford, and mHabitat

*Introduction*

This evidence-based guide has been developed from a systematic literature review and extensive primary research. It is designed to help you reflect on your ideas and goals for a **technology-supported change project** in health or social care and work towards a project plan. A high proportion of such projects fail, but there are ways of improving the chances that your project will succeed.

Technology projects are characterised by **complexity** – i.e. they have multiple interacting components that cannot be tightly controlled. Complex projects are unpredictable and risky, hence less likely to succeed than simple ones. This guide will help you to identify the different areas of complexity (that is, the uncertainties, interdependencies and possible unintended consequences) in your project and think of ways to reduce or manage these (e.g. by making some aspects simpler or mitigating risks)

*How to use this guide*

We recommend that you start using this guide as early as possible and keep revisiting it as your project unfolds. It will only take you a few minutes to skim through it and gain an initial orientation, but working carefully through the detail of the guide will take much longer. There is no ‘right’ way to use the guide; it is intended to prompt conversations and help you bring together different areas of expertise (such as clinical, technical and business development). For example, you could assign different parts of the guide to different people to fill in in detail, then reconvene and compare your responses. You may wish to employ a facilitator to run a workshop with the project team.

*Structure of this guide*

PART 1 of this guide is divided into 6 domains, each in two parts:

- **A free-text box for you to present this domain in your own words**. This will help surface the issues, technologies, people and activities relevant to *your* project and how they seem to fit together. Make it flow like a story (i.e. write in sentences rather than using tables or bullet points), so as to capture the messiness (non-linearity) of the project. Telling a brief story will allow you to draw out the ‘plot’ of what’s happened so far and identify interdependencies and tricky issues that may contribute to the project’s success or failure (or something in between).
- **Some** **questions to help you estimate key areas of complexity** (most of which should have come up in your narrative). The more red boxes you tick, the more complex this domain is (though the boxes don’t carry equal weight, so adding up the ticks won’t give you a quantitative score). The top-level questions are quite broad, but if a question is particularly relevant to your proposed project, you can ‘drill down’ with the more detailed questions. Ideally, you should be able to back up your answers with evidence, such as published figures or research, or data you have collected yourself (for example from interviews or focus groups). Some questions will not apply to your project, so tick ‘not applicable’ for these. If a question seems relevant but you’re not sure how to answer it, tick ‘don’t know’ – and perhaps discuss this one with colleagues later. Can you distinguish the things you don’t *yet* know (but could find out) from the things that are unknowable (inherently uncertain), which you have to handle with creativity and judgement as the project unfolds?

Note: [1] No single individual will be able to answer all the questions but you should find that if you involve a range of people across your organisation, you will be able to address all the domains. For each domain, we’ve suggested who might be best placed to answer the questions. [2] The tick-box questions will give an artificially structured and linear perspective. Bear in mind that complex change is an inherently messy and unpredictable process, but the box-ticking may help you find a ‘way in’ to your narrative.

PART 2 is designed to help your team handle the different kinds of complexity in your project. It consists of prompts to help you plan and manage an implementation project and think about how to

- **Reduce complexity** where possible (e.g. by limiting the scope of the project)
- **Respond to complexity** where it can’t be reduced (e.g. by bringing staff together to make sense of a situation, strengthening relationships, or collecting and analysing real-time data)

**
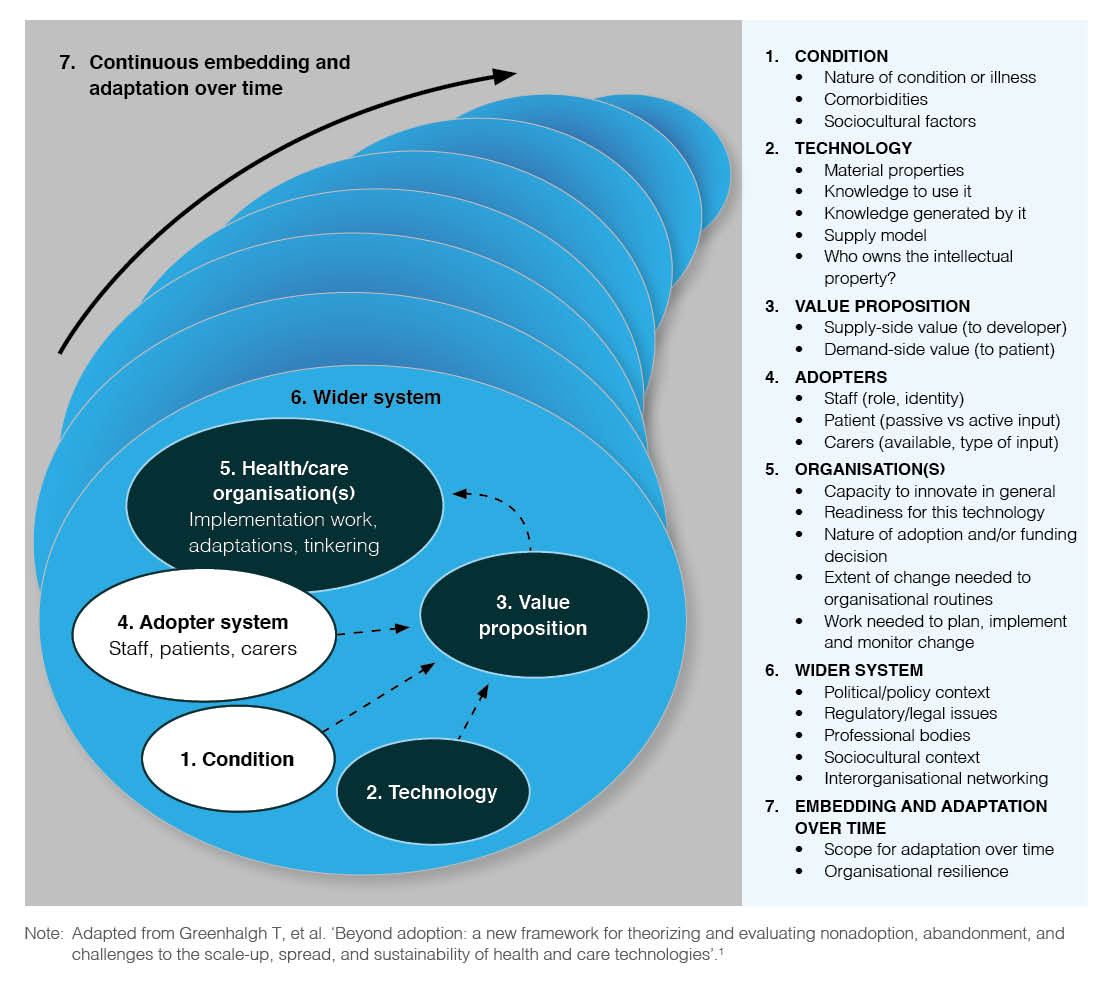
**

**Diagram: The NASSS framework** (© Greenhalgh at al [J Med Internet Research 2017; 19 (11): e367](https://www.jmir.org/2017/11/e367/))

# PART 1: ANALYSING COMPLEXITY IN YOUR PROJECT

## THE ILLNESS OR CONDITION [a clinician, social worker or researcher might be the best person to complete this section]


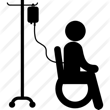
*Briefly describe the condition(s) for which the innovation or technology has been designed (e.g.
heart failure, mental health, social isolation). In some situations, there won’t be a specific illness
or condition.*

*The following questions should help you summarise whether the condition or illness is straightforward, well-understood, follows a predictable course and has predictable implications for care. This isn’t about whether the illness is* serious*, but whether you can predict what will happen next. For suggestions for responding to complexity in this domain, see page 13.*

| *IDENTIFYING COMPLEXITIES IN THE ILLNESS OR CONDITION:* | *Agree* | *Disagree* | | *Not applicable or don’t know* | | |
| --- | --- | --- | --- | --- | --- | --- |
| *There are significant uncertainties about the illness or condition*  *Additional detail – e.g.*   - *The condition is not clearly defined, or too little is known about it to inform planning* - *The population affected by the condition is not well-defined* - *The condition affects people in different ways, so a ‘one size fits all’ solution is unlikely to work* - *People with the condition are likely to be under the care of multiple professionals and/or in more than one care pathway* |  | |  | |  |  |
| *Many people with the condition have other co-existing illnesses or impairments that could affect their ability to benefit from the technology or service*  *e.g.*   - *Physical or mental co-morbidities* - *Cognitive impairment* |  | |  | |  |  |
| *Many people with the condition have social or cultural factors that could affect their ability to benefit from the technology or service.*  *e.g.*   - *Poverty* - *Social exclusion e.g. drug use, homeless* - *Religious restrictions or expectations on how they manage their illness* - *Low health literacy (limited ability to understand health issues and how to handle them)* - *Low system literacy (limited understanding of how services work and how to navigate them)* - *Low digital literacy (limited ability to use, or learn to use, new IT products)* - *Unable to understand the language used by professional staff* |  | |  | |  |  |
| *The population with the condition, and/or how the condition is treated, is likely to change significantly over the next 3-5 years* |  | |  | |  |  |
| SUMMARY: The illness or condition has significant complexity which is likely to affect the project’s success | | *Yes* | | *No* | | |

## THE TECHNOLOGY (or other innovation) [the technology developer might be the best person to complete this section]


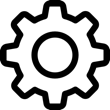
*Describe the technology/ies or other innovation. It might be an app, a device, a tool, a protocol
or pathway, an algorithm, a model, a piece of hardware – or some combination of these.*

*Highlight what is new apart from the technology (e.g. new way of working).
An innovation can be old technology (e.g. telephone) used in a new way.*

*The questions below will help you decide if the technology (and how it works to support care) is straightforward, well-understood and will have a predictable effect. For suggestions for responding to complexity in this domain, see page 13.*

| *IDENTIFYING COMPLEXITIES IN THE TECHNOLOGY OR OTHER INNOVATION:* | *Agree* | *Disagree* | | *Not applicable or don’t know* | | |
| --- | --- | --- | --- | --- | --- | --- |
| *There are significant uncertainties about what the technology is*  *e.g.*   - *The technology is difficult to define (e.g. connects with hidden infrastructure, supplier does not disclose full details)* - *The technology does not yet exist in a robust and definitive form* |  | |  | |  |  |
| *There are significant uncertainties about where the technology will come from*  *e.g.*   - *The technology supply chain is not yet in place* - *The technology is not easily substitutable (i.e. if the supplier withdrew, it would not be obtainable elsewhere)* |  | |  | |  |  |
| *There are significant uncertainties about the technology’s performance and dependability*  *e.g.*   - *Data collection and transmission (where relevant) are not yet accurate or reliable* - *There are significant privacy or security concerns* |  | |  | |  |  |
| *There are significant uncertainties about the technology’s usability and acceptability*  *e.g.*   - *It is not possible for people to try out the technology on a small scale before adopting it* - *The data or knowledge generated by the technology is not well understood or trusted* - *There is not yet evidence from prototyping that intended users find the technology easy to use without human support (e.g. clinician, carer or help desk)* - *There is not yet evidence from prototyping that the technology is acceptable to its intended users (e.g. that it generates data that are well-understood and trusted, and which reflect how their condition is normally managed)* |  | |  | |  |  |
| *There are significant technical interdependencies*  *e.g.*   - *A key technology needs to be installed across multiple technical systems so as to achieve ‘integration’* - *The technology cannot be installed until the organisation’s IT system is upgraded or changed (e.g. new hardware, better bandwidth)* - *The technology would require individual users to upgrade their device(s) or home IT system* - *The technology overlaps (unproductively) with an existing technology that performs the same or a similar function* |  | |  | |  |  |
| *The technology is likely to require major changes to organisational tasks and routines*  *e.g.*   - *Implementing the technology means some staff will have to do their jobs in a different way and/or interact with different people* - *Implementing the technology will require new or different steps in the overall care pathway (e.g. new administrative processes)* |  | |  | |  |  |
| *The technology (and/or the service model it supports) is likely to change significantly within the next 3-5 years*  *e.g.*   - *The technology has limited potential to be adapted to take account of future clinical developments and other changes* - *The technology supply model may not be sustainable (e.g. the client-supplier relationship is weak, or there are questions about the company’s reputation)* |  | |  | |  |  |
| SUMMARY: The technology has significant complexity which is likely to affect the project’s success | *Yes* | | *No* | | |  |

## THE VALUE PROPOSITION (costs and benefits of the technology) [the technology developer and business lead for the organisation might complete this section]


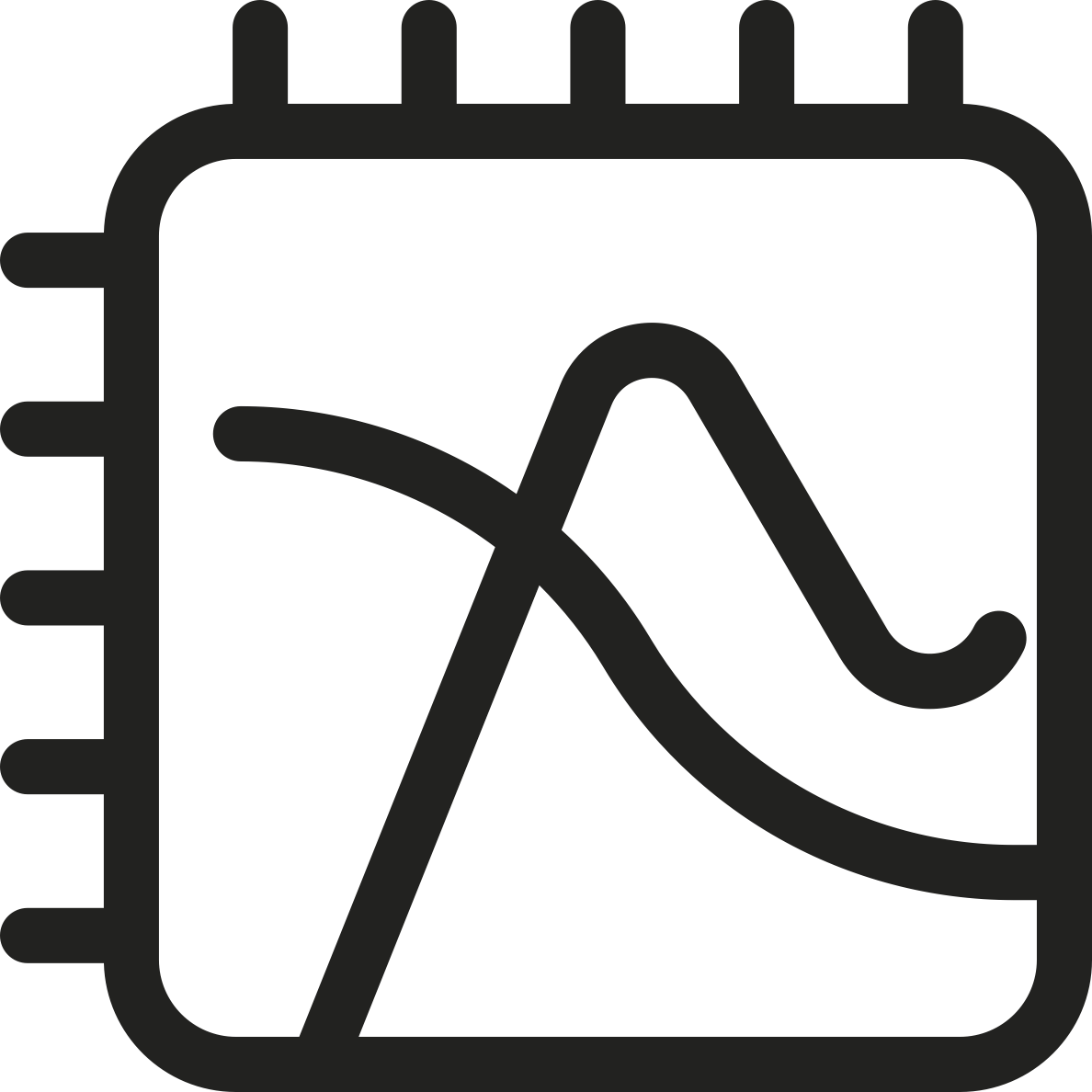
*Describe the value (financial or otherwise) that the new technology and care model might generate.
For commercial stakeholders, this may be return on investment. For patients, it may be cure,
comfort, or quality of life. For healthcare organisations, it may be improvements in quality of care,
efficiency (saving time, freeing up staff), safety (including reduced risk of litigation), or inclusivity.*

*The following questions address what kind of value the technology might generate for different groups of people. For suggestions for responding to complexity in this domain, see page 14.*

| *IDENTIFYING COMPLEXITIES IN THE VALUE PROPOSITION:* | *Agree* | *Disagree* | | *Not applicable or don’t know* | | |
| --- | --- | --- | --- | --- | --- | --- |
| *The commercial value of the technology is uncertain*  *e.g.*   - *If the technology does not yet exist in a definitive form, the case for investing in its [further] technical development is weak* - *The technology does not have a plausible business case, including up-front investment, a well-defined customer base and market drivers, consideration of competing products and realistic assessment of challenges of implementing at scale in a public-sector health or care environment* |  | |  | |  |  |
| *The value to the patient or client is uncertain*  *e.g.*   - *There are no high-quality studies (e.g. randomised controlled trials) to demonstrate the technology’s efficacy for this patient/client group* - *The technology’s benefits have not been shown to outweigh its potential harms* - *The technology’s efficacy and safety were not measured in terms of an outcome that matters to patients* |  | |  | |  |  |
| *The value to the clinician or other staff member is uncertain*  *e.g.*   - *The technology may create work (or other hassles) for the front-line staff* - *The technology’s benefits have not been shown to outweigh the hassle of using it* |  | |  | |  |  |
| *The value to the healthcare system is uncertain*  *e.g.*   - *The technology (or the technology-supported care model) is not considered to have any overall advantage over existing practice* - *The technology has not yet been shown to be effective and cost-effective in terms of how much benefit it will bring for a given financial outlay* - *There are safety concerns about the technology or care model* - *This technology-supported care model has not yet been successfully implemented in a similar context to the one being contemplated* - *There are concerns that the technology, whilst improving care for some patients, could widen inequalities* - *Regulatory and other approvals for the technology are not yet in place* |  | |  | |  |  |
| *The value to this particular healthcare organisation is uncertain*  *e.g.*   - *The technology will require new technical infrastructure before it can be introduced to this organisation (see Technology domain)* - *The technology will require extensive changes to organisational routines and pathways (see Technology and Organisation domains)* - *Aspects of the local procurement processes make it hard to commission this technology (see Organisation domain)* |  | |  | |  |  |
| *The technology could generate a negative value (i.e. costs are likely to outweigh benefits) for some stakeholders.*  *e.g.*   - *Potential loss of income* - *Destabilising a provider* - *Hidden or knock-on costs* |  | |  | |  |  |
| *The value proposition is likely to change significantly over the next 3-5 years.*  *e.g.*   - *A new, better technology is on the horizon* - *The market for the technology will change significantly*   *A key regulatory decision could be made or reversed)* |  | |  | |  |  |
| SUMMARY: The value proposition has significant complexity which is likely to affect the project’s success | *Yes* | | *No* | | |  |

## THE INTENDED ADOPTERS OF THE INNOVATION/TECHNOLOGY [this section should be completed by, or on behalf of, everyone who might use the technology]


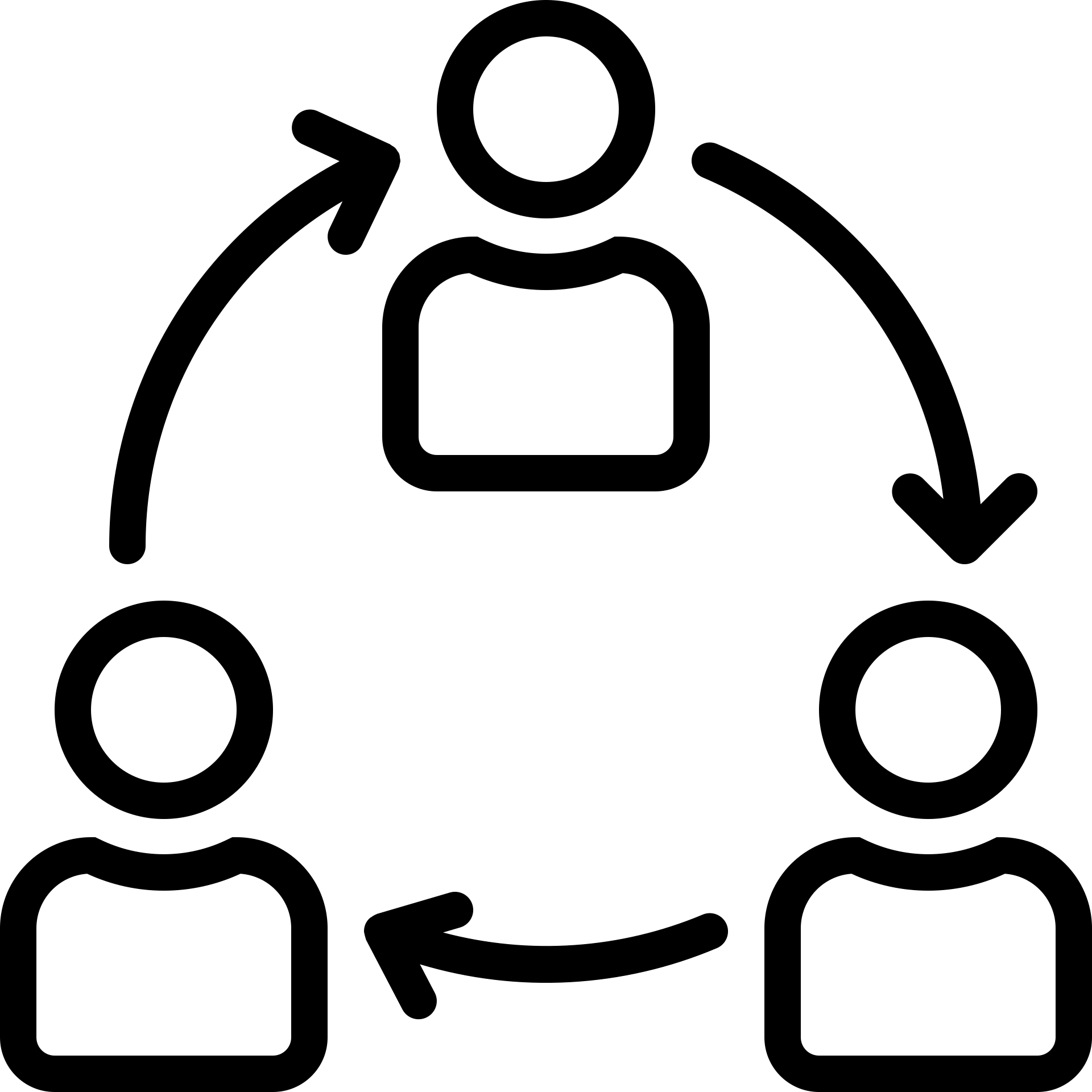
*Describe the intended users of the technology or other innovation. Consider: patients/lay people,
professionals, administrative and support staff. Are there people who will be impacted indirectly
(e.g. clinicians may be the main users but admin staff may need to adapt their procedures)?*

*The following questions will help you summarise whether people directly involved with the technology understand what it is for, think it is worth trying, feel able to use it and are motivated to give it a go, and also what the indirect knock-ons may be for others. For suggestions for responding to complexity in this domain, see page 14.*

| *IDENTIFYING COMPLEXITIES IN THE INTENDED ADOPTERS:* | *Agree* | *Disagree* | | *Not applicable or don’t know* | | |
| --- | --- | --- | --- | --- | --- | --- |
| *There is uncertainty about whether and how patients/carers or citizens will adopt the technology*  *e.g.*   - *The technology would require substantial input from the patient or their immediate carer* - *Some patients will view the technology in a negative way (e.g. not appropriate for their home, or reminding them of an illness they’d prefer to forget about)* - *Quite a few people in the intended user group may be unable or unwilling to learn to use the technology* |  | |  | |  |  |
| *There is uncertainty about whether and how front-line staff will adopt the technology*  *e.g.*   - *Some staff members question the value proposition for the technology (e.g. they feel that adopting it would jeopardise the quality or safety of patient care, or they believe it is more time-consuming than existing practice)* - *The technology would require staff to do their jobs differently, and perhaps take on a new, unwanted, role and identity (e.g. ‘data entry person’)* - *Some individuals or teams do not have the resources, time, space or support to learn to use the technology* - *Staff have not been trained or supported to be creative and flexible when implementing technologies* |  | |  | |  |  |
| *There is uncertainty about the implications for people who might be indirectly affected by the technology*  *e.g.*   - *The technology would require input from others (e.g. relatives, care home staff), who may be unable or unwilling to learn to use it* - *The technology would make someone else’s job obsolete or more difficult* |  | |  | |  |  |
| *There will be significant changes to individual users’ perceptions of the technology over the next 3-5 years*  *e.g.*   - *Key staff groups are likely to change their views on the technology* - *Patients or their lay carers are likely to change their views on the technology* |  | |  | |  |  |
| SUMMARY: There is significant complexity relating to the intended adopters which is likely to affect the project’s success | *Yes* | | *No* | | |  |

## THE ORGANISATION(S) IMPLEMENTING THE TECHNOLOGY [this section is best completed by people who know the organisation and the challenges it faces e.g. board member, human resources lead, staff representative]


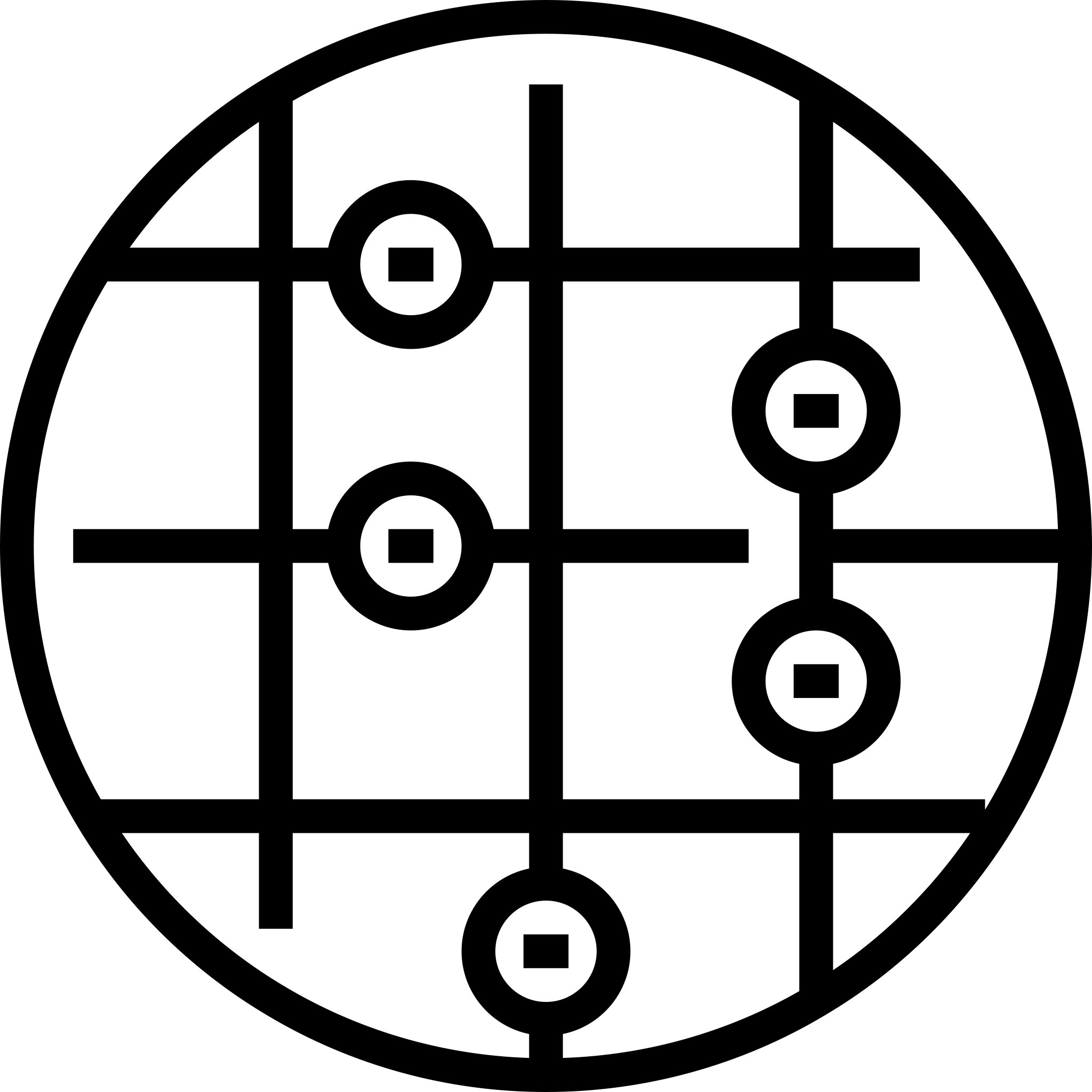
*Briefly describe the organisation(s) involved in the project (for example, digital agency, healthcare
provider, social care provider). What kind of organisation is it? How is it structured – and what is it
like to work there? What is its track record of taking up new technologies? How well-resourced is it*

*(in terms of both staff and funding)? Is there much enthusiasm for this particular technology?
You may need to complete this section separately for the main and partner/ impacted organisations (and use the highest complexity score in your planning, since the initiative will only be as strong as its weakest link).*

*The following questions will help you assess whether the organisation is capable and ready to take on the innovation, and whether the work involved has been understood and planned for. For suggestions for responding to complexity in this domain, see page 15.*

| *IDENTIFYING COMPLEXITIES IN THE ORGANISATION(S):* | *Agree* | *Disagree* | | *Not applicable or don’t know* | | |
| --- | --- | --- | --- | --- | --- | --- |
| *The organisation’s capacity to take on technological innovations is limited*  *e.g.*   - *Leadership is weak and the organisation’s mission and values are unclear* - *Internal relations, especially between managers and clinicians, are poor* - *The structure is top-down and hierarchical, so individual departments are discouraged from horizon-scanning for new products and ideas, and have limited scope to introduce innovations* - *The organisation has a poor track record of introducing any kind of change* - *There are no slack resources (people or money) to channel into innovative projects* - *It is not a learning organisation: staff are not encouraged to meet and talk about new ideas and projects, there are few or no measures in place to capture data and monitor progress, and risk-taking is discouraged* - *Digital maturity is low* |  | |  | |  |  |
| *The organisation is not ready for this particular innovation*  *e.g.*   - *The fit between the organisation’s mission and the innovation is poor* - *Key people (especially senior management) oppose the innovation or are unconvinced of its value* - *The business case is weak or questioned (see Value Proposition domain)* - *The implications (e.g. work required) of introducing, implementing and evaluating the technology have not been adequately assessed (or have been questioned)* - *Money is needed but a budget line has not been allocated* |  | |  | |  |  |
| *Organisational routines and processes will need to change very considerably to accommodate the technology*  *e.g.*   - *Different kinds of staff (e.g. new hires) will need to be involved in the process or pathway once the technology has been introduced* - *A new (or radically revised) process or pathway will need to be developed* - *The core process or pathway will need to link differently with other key processes and pathways in the organisation* |  | |  | |  |  |
| *Procurement processes are in place that make it harder to commission this technology*  *e.g.*   - *The provider is not on the procurement framework* - *Existing contracts need to expire first* - *Aspects of the procurement process are not yet clear (e.g. Who will fund this? Who will be liable for costs? Is there an identified budget? It is capital or revenue ? Is the funding recurrent? Are there issues with timing/accruals of funding?)* |  | |  | |  |  |
| *The work needed to introduce and routinise the innovation has been underestimated and/or inadequately resourced*  *e.g.*   - *Work to bring people on board and develop a shared, organisation-wide vision for the change* - *Work to develop, implement and mainstream new care pathways and processes* - *Work to coordinate the project across more than one organisation or sector* - *Work to evaluate and monitor the change* |  | |  | |  |  |
| *The organisation(s) involved are likely to have significant restructurings or changes in leadership, mission or strategy over the next 3-5 years* |  | |  | |  |  |
| SUMMARY: There is significant complexity relating to one or more participating organisations which is likely to affect the project’s success | *Yes* | | *No* | | |  |

## THE EXTERNAL CONTEXT FOR INNOVATION [this section might be completed by a ‘horizon-scanner’ who looks beyond the organisation]


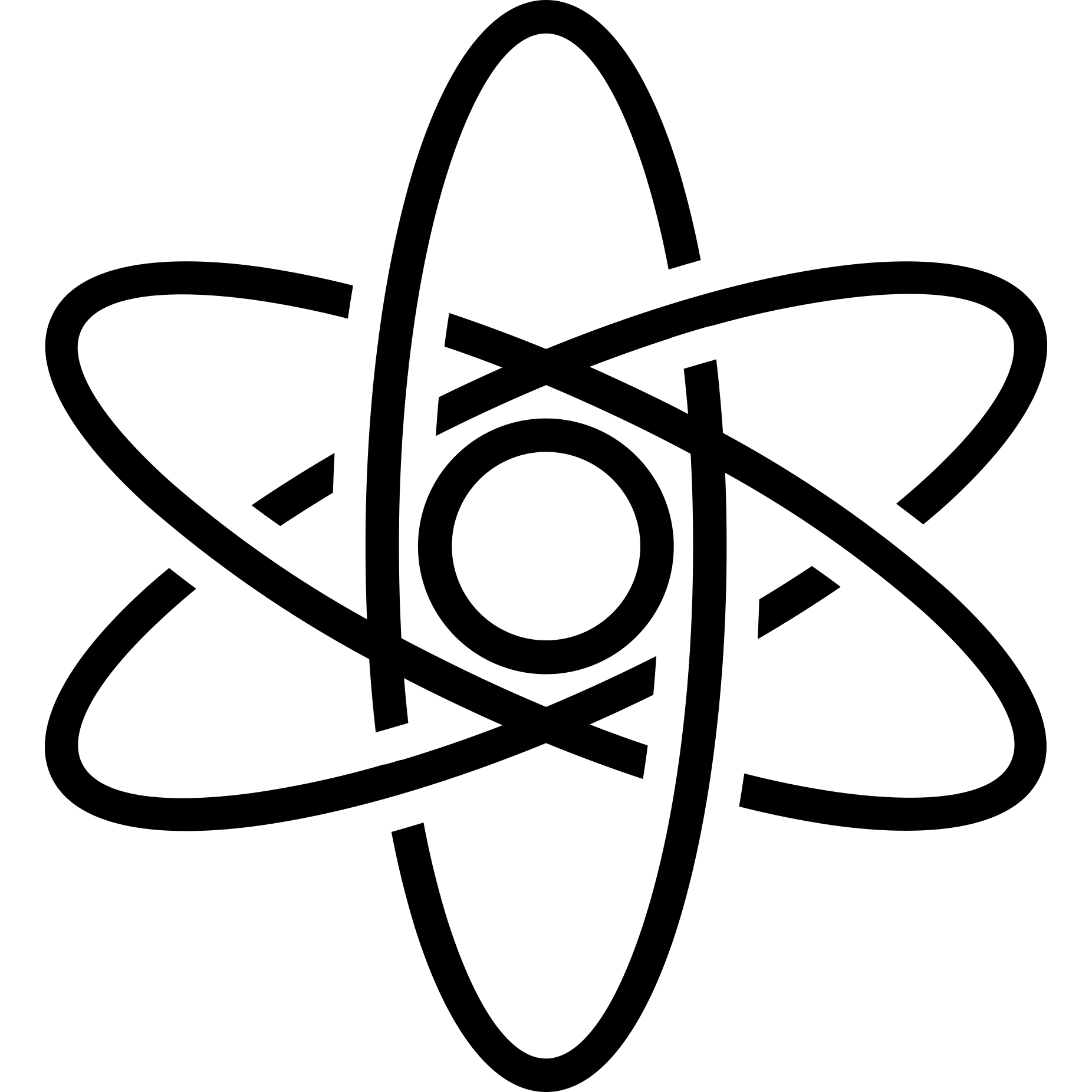
*Describe the national and local context for your technology or programme (e.g. legal obligations,
policy, professional bodies views on best practice, related national initiatives). Think about the
key influences on the project beyond the organisation(s) you identified in the previous section.*

*The following questions will help you summarise whether there are external conditions (such as the state of policy, public/ professional opinion, expected external events such as political climate change) likely to complicate the adoption and mainstreaming of the innovation. For suggestions for responding to complexity in this domain, see page 17.*

| *IDENTIFYING COMPLEXITIES IN THE EXTERNAL CONTEXT:* | *Agree* | *Disagree* | | *Not applicable or don’t know* | | |
| --- | --- | --- | --- | --- | --- | --- |
| *The political and/or policy climate is adverse*  *e.g.*   - *External political or economic changes impacting on the organisation could threaten the introduction of the innovation* - *Current policy priorities conflict with this initiative* |  | |  | |  |  |
| *Professional organisations are opposed to the innovation or don’t actively support it*  *e.g.*   - *There are concerns about quality or safety of care* - *There are concerns about confidentiality and wider information governance* - *There are concerns about professional workload* - *Priorities are elsewhere* |  | |  | |  |  |
| *Patient organisations and lobbying groups are opposed to the innovation or don’t actively support it*  *e.g.*   - *There are concerns about quality or safety of care* - *There are concerns about privacy and/or what will happen to the data* - *Priorities are elsewhere* |  | |  | |  |  |
| *The regulatory context is adverse*  *e.g.*   - *Quality standards and regulatory requirements for using the technology in a health or care setting have not been fully defined* - *Key stakeholders do not know about or accept these standards and requirements* |  | |  | |  |  |
| *The commercial context is adverse*  *e.g.*   - *The technology industry views the innovation (or similar products) negatively* - *The technology does not use industry-standard components* - *There is lack of support for timely updates to the technology to support ongoing work as intended* |  | |  | |  |  |
| *Opportunities for learning from other (similar) organisations are limited*  *Additional detail*   - *No other similar organisations are yet using the technology* - *Inter-organisational knowledge exchange networks are weak* |  | |  | |  |  |
| *Introduction of the technology/innovation could be threatened by external changes that impact on the organisation* |  | |  | |  |  |
| *The policy, regulatory and economic context for this innovation is likely to be turbulent over the next 3-5 years*  *e.g.*   - *Change of government* - *New policy priorities* - *Economic recession* - *New regulatory framework* - *Withdrawal of industry commitment* |  | |  | |  |  |
| SUMMARY: There is significant complexity relating to the external context which is likely to affect the project’s success | *Yes* | | *No* | | |  |

## EMERGENCE OVER TIME
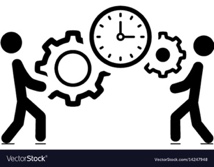
 [this section pulls together the bottom row of each of the previous domains]

*Summarise the main changes which, if they happen, could affect the project over the next
3-5 years. Which of these do you think is most significant? What are the key uncertainties?*

*For suggestions for responding to complexity in this domain, see page 18.*

| *ESTIMATING WHAT THE FUTURE HOLDS:* | *Agree* | *Disagree* | | *Not applicable or don’t know* | | |
| --- | --- | --- | --- | --- | --- | --- |
| *The population with the condition, and/or how the condition is treated, is likely to change significantly over the next 3-5 years* |  | |  | |  |  |
| *The technology (and/or the service model it supports) is likely to change significantly over the next 3-5 years* |  | |  | |  |  |
| *The value proposition for the technology is likely to change significantly over the next 3-5 years* |  | |  | |  |  |
| *There will be significant changes to individual users’ perceptions of the technology over the next 3-5 years* |  | |  | |  |  |
| *The organisation(s) involved are likely to have significant restructurings or changes in leadership, mission or strategy over the next 3-5 years.* |  | |  | |  |  |
| *The policy, regulatory and economic context for this innovation is likely to be turbulent over the next 3-5 years* |  | |  | |  |  |

# PART 2: ACTION PLANNING AND PROJECT MAGAGEMENT

Taking account all your responses to Part 1, this section prompts you and your team to **plan your implementation project** and consider measures to **reduce or respond to complexity** in the different NASSS domains. Below, we offer some ideas and resources to get you started. The resources and links have been selected for a UK setting but could easily be adapted for other countries.

**Planning your implementation project**

**Skim this section first – but then go on to look at the different complexities and ideas for responding to them. You may end up deciding not to go ahead with the project at all.**

Project management in a highly predictable environment is fairly straightforward, but under conditions of complexity, things can’t be fully predicted or laid out in advance. You need to set a broad goal, take action on several fronts simultaneously (making sure you attend to the human and political aspects of the project as well as the technical and financial aspects), while periodically reviewing progress and adjusting your strategy.

For large, ambitious projects, we recommend the [Project Initiation Routemap](https://assets.publishing.service.gov.uk/government/uploads/system/uploads/attachment_data/file/529311/handbook_2016.pdf), a guide by the UK government for planning complex projects in the public sector. The Routemap emphasises three linked strategic tasks:

Assess the complexity and context of the delivery environment (see NASSS questions above, especially Domain 2 ‘The Technology’ and Domain 5 ‘The Organisation’), and consider how you could respond to this complexity (see suggestions below);

Assess the capacity and capability of organisations and teams to deliver the project (in particular, sponsor, senior management buy-in and support, dedicated delivery team);

Work to strengthen and align context and capability (e.g. align requirements, governance, execution strategy, organisation design and development, procurement, risk management, asset management).

See also the [NESTA DIY toolkit for bringing ideas to life](https://media.nesta.org.uk/documents/diy-toolkit-full-download-a4-size.pdf) (designed for social care providers) – a structured way to get from the initial idea for a new technology to a well-designed project to get it up and running in a service.

Due diligence. Before investing in a technology, make sure the company selling it is legal and solvent, that the technology has the requisite regulatory approvals, that personal data is handled sensitively and respectfully, and that any associated risks have been considered. There are numerous due diligence checklists available – see these for example:

[UK government digital service standards](https://www.gov.uk/service-manual/service-standard) – a 14-point checklist when planning a service that involves digital technology. Linked to these are [UK government technology and digital standards](https://www.gov.uk/service-toolkit).

[How to do due diligence for health care technologies](https://www.securedocs.com/blog/how-to-do-due-diligence-for-healthcare) – introductory blog from private company SecureDocs.

[Digital Assessment Questionnaire](https://developer.nhs.uk/wp-content/uploads/2018/09/Digital-Assessment-Questions-V2.1-Beta-PDF.pdf) from NHS Digital, a self-assessment checklist for apps and similar technologies.

[Medical devices – software applications](https://www.gov.uk/government/publications/medical-devices-software-applications-apps) – Advice from the UK government on when software applications are considered to be a medical device and how they are regulated.

NHS Health and Social Care [Data Security Standards](https://www.google.co.uk/url?sa=t&rct=j&q=&esrc=s&source=web&cd=1&ved=2ahUKEwi90o69gsbjAhXQSxUIHTPBD2YQFjAAegQIBRAB&url=https%3A%2F%2Fwww.dsptoolkit.nhs.uk%2FHelp%2FAttachment%2F125&usg=AOvVaw05CPQr_2TQ6V2vyk5OFT_K) (including a full due diligence checklist for suppliers).

[UK government code of conduct for data-driven health and care technology](https://www.gov.uk/government/publications/code-of-conduct-for-data-driven-health-and-care-technology/initial-code-of-conduct-for-data-driven-health-and-care-technology) – Principles and advice for machine learning applications that use NHS data.

Commercialising new technologies. If you are developing a new technology and you think it has commercial potential, you will need to systematically demonstrate to investors how it will generate value. Try this resource:

[Guidance and Impact tracking System (GAITS)](https://www.gaits.org/) – a web-based project and portfolio management platform designed to support commercialisation of new health technologies, developed by the US consultancy firm CIMIT.

[Adoption Readiness Level tool](https://www.ehealthcluster.org.uk/arl-tool/) by Liverpool City Region’s e-health cluster – a self-assessment tool for tech developers that considers five domains (market, human, systems integration, finance/procurement, motivation).

**Responding to complexity in the** **illness or condition**

**Your first step in developing technological solutions for an illness or condition is to understand the full range and depth of what the illness *is* and how it affects people.**

Find out more about the illness. For example, find the prevalence, likely progression, and current ‘best practice’ care model. This will allow you to estimate how many users a product is likely to have, how long they can/will use it for, and how this fits with current care. Remember, there will be ‘mild’ and ‘severe’ forms of the illness, different age groups, ethnicities, genders and so on. Once you understand how the illness is patterned, this could inform work to ‘personalise’ the technology for different sub-groups (see ‘Responding to complexity in the intended users’ below). To learn about the illness, use different data sources, e.g. from national and regional databases, academic and grey literature, health and care practitioners, patient organisations, patients. For example:

[NHS Choices](https://www.nhs.uk/conditions/) – a searchable database of illnesses, including diagnosis, treatment and likely course

[NICE guidelines](https://www.nice.org.uk/guidance) - evidence-based recommendations in a variety of conditions, procedures and technologies across health and social care developed by independent committees

[Cochrane library](https://www.cochranelibrary.com/) – a database of high-quality systematic reviews of treatments

[Healthtalk](http://www.healthtalk.org/) – a database of patients’ accounts of what it’s like to live with different illnesses

[Macmillan](https://www.macmillan.org.uk/) – a website for people with cancer, with detailed information on prevalence, treatment and prognosis. There are similar patient-facing websites for most conditions. Explore them!

**Responding to complexity in the** **technology(ies)**

**Don’t make the mistake of treating a new technology as a plug-and-play solution. You need to ask a lot of questions about it before you can be sure it’s the right tool for the job. New technologies often look appealing and promising until we consider all aspects of the innovation process.**

Find out more about the technology and assess its quality and implications. If you are not the creator of the technology, familiarise yourself with all relevant aspects of it or ask an expert. Look at it; play with it; do a ‘walk through’ the imagined use case. Will this product really help with what you are planning to achieve? Could a different technology (perhaps one that is already tried and tested) do a similar job with less hassle?

[NHS apps library](https://www.nhs.uk/apps-library/) – a searchable database of quality-assured smartphone health apps

Publicly available ‘curated’ databases of apps – for example:

[Psyberguide](https://psyberguide.org/) for mental health apps

[ORCHA](https://www.orcha.co.uk/), an independent organisation that evaluates apps

Find out more about where the technology will come from and associated challenges. Ideally the building blocks for your chosen technology e.g. coding platform, devices etc can be accessed or purchased easily (no long waiting periods or unreliable supply chains). Ideally, the technology should not depend on a single vendor/device/coding language etc, but work (or have the potential to work with or easily change to) others as well. They will have been tested extensively so you don't have to worry about these components being dependable. Conflicts of interest and claims to intellectual property (IP) should be sorted out before the project begins. It should be clear who will fund the technology, what it will cost and which costs are covered (set up, maintenance, updates etc).

Identify and address the key points where technical complexity will impact on success. Find out about any unknowns and dependencies as soon as possible, and develop a plan to deal with them, including alternatives or workarounds. Reduce unnecessary technical integration. Integration between multiple systems makes everything more complex. Ask whether it is really necessary or if there are ways to avoid or delay this, especially during initial testing. But bear in mind that some forms of technical integration (e.g. to make a new piece of software accessible from within a patient's existing electronic record) may make the technology *simpler* for a clinician to use.

Consider how the technology will disrupt the system. Map possible disruptions and take steps to avoid or mitigate them. Can you modify the technology to make it less disruptive? Can you reduce knock-ons by adjusting other systems or processes? What measures might you put in place (e.g. small-scale pilot running in parallel with the old service, on-the-job training, help desk) to deal with the disruption until systems and processes have evolved to accommodate the new technology? We pick up this important point again under ‘the organisation’ below.

**Responding to complexity in the** **value proposition**

**This project is only going to work if all stakeholders gain something of value from it.**

Consider how to increase the technology’s appeal to investors. If the technology is at an early stage of development, what is its likely upstream value as viewed by investors (especially the business case for generating profits, further spin-offs, and highly qualified jobs), drug and device regulators (preliminary evidence of efficacy and safety), and financial regulators (auditable business processes and governance)? Can the technology be ‘de-risked’ by removing costly but inessential features? See the [Guidance and Impact tracking System (GAITS)](https://www.gaits.org/) resource linked above.

Consider how to increase the technology’s value to patients or citizens. If a technology is meant to be used by patients or lay people, its potential benefits must be weighed against its costs (and the person’s willingness and ability to contribute to these), the work needed to use it (and whether the person or their carer is able and willing to do that work), and the desirability of medicalisation and surveillance. Can the design be improved to make the technology more appealing? Can the data be visualised in a way patients or carers can engage with?

See links above under ‘Responding to complexity in the illness’

[Getting the most out of PROMS](https://www.kingsfund.org.uk/sites/default/files/Getting-the-most-out-of-PROMs-Nancy-Devlin-John-Appleby-Kings-Fund-March-2010.pdf) – A guide to using patient-reported outcome measures to assess whether an intervention or technology is actually improving outcomes that are valued by patients

[A guide to PROMs methodology](https://digital.nhs.uk/binaries/content/assets/legacy/pdf/g/t/proms_guide_v12.pdf) from NHS Digital (using hip and knee replacement as an example)

Identify evidence of effectiveness and cost-effectiveness. If the technology is at a more advanced stage of development, there may be research evidence comparing its effectiveness (does it work?) and cost-effectiveness (is it good value for money?) with ‘usual care’ and measuring an outcome that is important to patients. Try these resources:

[NICE Evidence Standards for digital health technologies](https://www.nice.org.uk/about/what-we-do/our-programmes/evidence-standards-framework-for-digital-health-technologies) – These cover both effectiveness and economic impact.

Consider real-world value issues. Is there a realistic assessment of the challenges of implementing this innovation at scale in a particular public-sector health or care environment? Even when something has been shown to be cost-effective, it may not be locally affordable or a funding priority.

The NICE Evidence Standards website linked above offers a [budget impact guide](https://www.nice.org.uk/Media/Default/About/what-we-do/our-programmes/evidence-standards-framework/budget-impact-guide.pdf) and [budget impact template](https://www.nice.org.uk/Media/Default/About/what-we-do/our-programmes/evidence-standards-framework/budget-impact-template.xlsx) for local cost planning.

**Responding to complexity in the intended** **adopters of the technology**

**This project is only going to work if the people you want to use the technology are able and willing to do so.**

Address acceptability, accessibility and usability for patients and citizens. If the technology requires input from a patient, carer or other lay person, will they find the product aesthetically pleasing and easy to use? Does the technology make sense, for example, in the context of how patients and carers already do things, their routines and existing tools they use to support their work? Remember, everyone is different. Some people have limited vision or dexterity; some people find instructions hard to understand. Can you make the product more accessible? Is it worth building design changes in now or planning to do so in the future (e.g. after proof of concept testing)? If the technology includes several components, can users select what is most relevant for them? These resources may help:

How to do research on user needs in the [‘discovery phase’](https://www.gov.uk/service-manual/user-research/user-research-in-discovery) of technology design – a website from the UK government.

[International Design Foundation](https://www.interaction-design.org/literature/article/accessibility-usability-for-all) – a US site offering tips and resources for making websites and apps more accessible.

[How to design websites for older people](https://www.alzheimers.org.uk/blog/how-design-websites-older-people) – a guide from the Alzheimers Society.

Address staff motivation and concerns. Assess the level of enthusiasm for the technology from different staff groups, and also how motivated teams are to take on the new technology. Have any of them had experience of using this technology elsewhere? Listen to staff concerns – which may be legitimate – and to their ideas for increasing the project’s success. This resource may help:

Higher Education England [Digital Capabilities Framework](https://www.hee.nhs.uk/sites/default/files/documents/Digital%20Literacy%20Capability%20Framework%202018.pdf) for assessing the digital capability of staff.

Modify staff roles and provide training. Develop new roles and job descriptions where needed, perhaps by adapting ones already in use elsewhere. Set learning objectives (some of which will be about building confidence to make judgements, not about mechanically following protocol). Design and develop training courses. Remember: using a technology usually needs on-the-job and team-based training, not just sitting in classrooms. Allocate sufficient budget for this work, and consider issues such as backfill.

Promote social learning. One way to become confident in using a technology is to shadow someone in the same role who is already an enthusiast for it (‘champion’) and confident in using it (‘super user’). Learning in this way not only develops skills but also helps people develop a positive attitude and identity.

Support collective sensemaking and communities of practice. People need to make sense of new technologies – sometimes by coming together to complain about them initially! Surfacing one’s irritation with a technology may be the first step to coming to terms with it. Both staff and patients may benefit from being in ‘communities of practice’ (groups or networks of people who share an interest in something and are trying to get better at it). Online communities of patients, for example, are often good sources of knowledge and wisdom about how to manage a condition. Try to get these communities on board if introducing a patient-facing technology.

The [Kings Fund guide](https://www.kingsfund.org.uk/sites/default/files/field/field_publication_file/staff-engagement-feb-2015.pdf) to engaging NHS staff may provide some practical ways of achieving the above.

**Responding to complexity in the** **organisation**

**The project is only going to work if the organisation has the capacity to take on innovations and if there is good ‘innovation-system fit’. The tips below may help if you are trying to support an organisation to implement a new, technology-supported care model.**

Assess the organisation’s capacity to innovate. An innovative organisation has strong leadership, good clinician-managerial relations, a devolved management structure, slack resources (money and/or staff) that can be channelled into new projects, good lines of communication and an ethos where it’s OK to take risks and learn from failures. If an organisation appears to lack these essential prerequisites for innovation, consider whether you need to strengthen its capacity before pressing ahead. Here are some questions to help you assess capacity to innovate:

Is there a culture that supports innovation and change (e.g. are staff trusted to introduce new ideas)?

Does the organization have systems and processes in place that support innovation and change e.g. effective information and communication systems, opportunities for networking and learning across departments/teams?

Do the senior management team actively seek opportunities for improvement and encourage ideas and feedback from patients, the public and staff?

Are the organisation’s leaders helping to create a facilitative context through providing motivation and support, creating a vision and reinforcing the change process?

Is there a distributed and devolved style of management?

Is there a history of introducing successful change in comparable projects at a local level?

Are there mechanisms in place to support learning and evaluation and to embed changes in routine practice e.g. regular team meetings, audit and feedback processes, professional development opportunities and performance review systems?

Assess innovation-system fit. Even when an organisation is capable of running a successful project to implement a new technology, it might be the wrong technology to introduce in this organisation right now. Has the organisation successfully adopted similar technologies in the past? Are its strategic priorities aligned with the use of the proposed technology? Or are other projects more pressing?

Assess the implications of the technology for the organisation. Careful mapping out of tasks and processes is necessary to surface how the technology or other innovation is likely to change these. The pathway in which the technology is used directly (e.g. clinical care) may have indirect knock-ons for other processes and pathways (e.g. booking, correspondence, billing). You need to estimate costs (both initial and recurrent), and consider how money will need to flow across the system. Before signing off on a project, boards generally want to know how much will it cost up-front, what the likely savings will be, and when these savings will occur. These resources may help:

[Process mapping guide](https://improvement.nhs.uk/documents/2143/conventional-process-mapping.pdf) from NHS Improvement. Ideas and tools for mapping the steps in a care pathway. A full list of additional service improvement and redesign tools from NHS Improvement is available [here](https://improvement.nhs.uk/resources/quality-service-improvement-and-redesign-qsir-tools/#project).

[Using costing information to support better outcomes](https://improvement.nhs.uk/resources/using-costing-information-to-support-better-outcomes/) – a guide from NHS Improvement.

Assess the level of ‘political’ backing for the innovation. For an organisational-level adoption decision to be approved, it needs support from both top management (a ‘senior sponsor’) and the rank-and-file. Supporters of the change must outnumber opponents and be more strategically placed. People with ‘wrecking power’ can block progress and may need to be brought on board (or worked around). To assess all this, use the NASSS-CAT PROJECT tool and also:

[Stakeholder analysis guide](https://improvement.nhs.uk/documents/2169/stakeholder-analysis.pdf) from NHS Improvement. This guide will help you construct a table or chart listing all the stakeholders who will need to accept (and, in many cases, start to use) the technology. Consider each key stakeholder’s perspective (and their potential wrecking power).

Consider inter-organisational relationships. Costs and benefits of technology projects are hard to predict, and savings may accrue elsewhere in the system. When there is no pre-existing contractual relationship between organisations, it can be hard to reach a satisfactory arrangement for how to manage these uncertainties.

Think how (and by whom) success will be evaluated. If this project is going to happen, you will need to monitor how well the change is going. You will almost certainly need both quantitative metrics (to answer the “how many…?” and “are we on track…?” questions) and also qualitative measures (to answer the “how do people feel about this…?” questions). Evaluation is everyone’s job, and data are often best collected by people doing the job. Extensive data collection can be time-consuming and slow the project down (i.e. the perfect may be the enemy of the good).

[Evaluation: what to consider](https://www.health.org.uk/sites/default/files/EvaluationWhatToConsider.pdf) – A guide by the Health Foundation. This basic guide includes qualitative and quantitative approaches.

[The ‘rainbow framework’ for evaluation and monitoring](https://www.betterevaluation.org/rainbow_framework) by Michael Quinn Patton. It takes you through 7 colour-coded steps, namely Manage (e.g. define stakeholders, secure funding), Define (set a scope for the evaluation), Frame (intended users of the evaluation, what they will use it for, what success will look like), Describe (sample, measures/metrics, data sources, analytic approaches), Understand Causes (deeper analysis to produce explanatory models), Synthesise (combining results), and Report & Support Use (publishing and disseminating).

[Evaluation Works and Evidence Works](https://www.weahsn.net/our-work/evidence-informed-commissioning/evidence-and-evaluation-toolkits/) toolkits to guide commissioning decisions, produced by West of England Academic Health Sciences Network and their partners.

Allocate funding. Studies of ‘failed’ technology projects often identify inadequate funding as a leading cause. You will probably need substantial set-up funding and possibly a recurrent budget line (for things like licences and IT support). Budget adequately for staff to learn and adjust as the transition occurs (see ‘Responding to complexity in the intended adopters’ above).

Manage the transition. Good change management involves a combination of ‘hard’ and ‘soft’ approaches. As well as setting goals and milestones and using agreed metrics to monitor progress, you also need to create opportunities for staff to come together and talk about the technology and new care model. As noted above, collective sensemaking, training (especially on-the-job training for both individuals and teams) and social learning from champions and super-users is crucial for building capacity. Use creative tools such as flip-charts and post-it exercises to surface people’s interpretations and concerns. Invite them to come up with creative ideas and solutions to any problems they identify. Allocate sufficient budget for this work, and consider issues such as backfill. This guide may help:

[Leading large-scale change: a practical guide](https://www.england.nhs.uk/wp-content/uploads/2017/09/practical-guide-large-scale-change-april-2018-smll.pdf) from NHS England.

**Responding to complexity in the** **external environment**

**Plans for technology-supported change locally are unlikely to work out if there is a major mis-match with national policy or the prevailing political, economic or professional environment.**

Try to align your project with current policy priorities. If the technology is actively supported in policy, it will be easier to introduce. If priorities are elsewhere, it may be worth trying to ‘rebrand’ the work to fit these.

Address regulatory issues and challenges. Consider which regulations (from which regulatory bodies) are relevant to the introduction of this technology. Are all approvals already in place? If not, who do you need to work with to make progress in this regard? See ‘Due diligence’ section on page 12.

Get the professions on board. If clinicians or social workers believe that the technology compromises the care of their patients or clients, or if they view it as demeaning to their role or a threat to their professional jurisdiction or income, their professional bodies may oppose it. Early dialogue with such bodies may avert such a situation.

Establish inter-organisational networks or collaboratives. Complex, organisation-wide change is a lot easier if change agents in one organisation can network with their opposite numbers in comparable organisations – for example in quality improvement collaboratives or learning sets. Here’s a resource for that:

[Improvement collaboratives in health care](https://www.health.org.uk/sites/default/files/ImprovementCollaborativesInHealthcare.pdf) – A guide from the Health Foundation.

Keep a close eye on the outer context. External shocks to an organisation (such as economic turbulence) make change precarious. Whist such shocks are often hard to predict, it is a good idea to see what’s on the horizon. The following questions may help you:

Does the new technology and the proposed changes to services align with the strategic priorities for the wider health system e.g. in terms of current health policy, national priorities for action and improvement?

Are there incentives in the wider health system that reinforce the proposed change e.g. pay for performance schemes, regulatory requirements etc.?

Are there existing inter-organisational networks (e.g. specialised clinical networks) that will be helpful in terms of supporting the proposed changes?

How much stability/instability is there in the wider health system – and how might this likely influence the implementation project?

**Responding to** **emergent complexity (new complexities that develop over time)**

The point about emergent change is it’s difficult if not impossible to predict. So this domain is really about how you might build resilience in your staff and your organisation to enable them to respond to things that come up in the future.

Acknowledge unpredictability. Have you left open the possibility that the project might unfold in one of several different ways? Can you flesh out these different possible futures and talk them through with your stakeholders?

Recognise and support self-organisation. Front-line teams will ‘tinker’ – that is, try to adapt the technology and the work process to make them work better locally. Are you able to capture data to evaluate and support these efforts?

Facilitate interdependencies. Have you identified the key interdependencies in the project? Is there anything you can do to strengthen existing interdependencies or develop and strengthen new ones?

Maintain space for experimentation and sensemaking. As complex projects unfold, staff will need to tinker more, and also talk about what’s happening. Encourage them to admit ignorance, explore paradoxes, exchange different viewpoints (there’s no need for them to agree on a single version of the ‘truth’!) and reflect collectively.

Develop adaptive capability in staff and teams. Train your staff to be creative and to adapt to change as it happens. They will sometimes need to make judgements in the light of incomplete or ambiguous data.

Attend to human relationships. Dealing with emergent problems requires give-and-take. It’s sometimes a matter of muddling through. This will happen more easily if people know, like and trust each other.

Harness conflict productively. There is rarely a single, right way of addressing a complex problem, so view conflicting perspectives as the raw ingredients for producing multifaceted solutions.
