# Supplementary material for: The NASSS-CAT Tools for Understanding, Guiding, Monitoring, and Researching Technology Implementation Projects in Health and Social Care: Protocol for an Evaluation Study in Real-World Settings
Source: JMIR Res Protoc. 2020 May 13;9(5):e16861. doi: 10.2196/16861 (PMC7254278; doi:10.2196/16861)
Supplement: Multimedia Appendix 4 [file resprot_v9i5e16861_app4.docx]

**NASSS-CAT (PROJECT VERSION)**

**FOR MONITORING PROJECT COMPLEXITY OVER TIME**

*This version of the NASSS-CAT is intended to be used when you are setting up and running a specific project to implement a new technology in a health or care setting. You may be asked to complete it more than once as the project unfolds. Score one point for every ‘agree’ answer and add up the orange column. In the blue column, tick if you think this issue is going to get more complex in the next phase of the project. Note: this tool will only give you a semi-quantitative estimate because some aspects of a project will be more important than others.*

|  | *Agree* | *Disagree* | *Not applicable*  *or don’t know* | *Likely to get more complex in next phase* |
| --- | --- | --- | --- | --- |

| *STRATEGIC COMPLEXITIES* | | | | |
| --- | --- | --- | --- | --- |
| 1. The vision and benefits for the project are not yet clear |  |  |  |  |
| 1. The fit between this technology and the organisation’s mission and strategy is poor |  |  |  |  |
| 1. The business case for the work is unclear or contested |  |  |  |  |
| 1. The scope of the project is unclear or contested |  |  |  |  |
| 1. The work will have major knock-ons for other key projects and business-as-usual operations |  |  |  |  |
| 1. Success criteria are not yet explicitly set out and agreed by key stakeholders |  |  |  |  |
| 1. The project’s success could be threatened by external changes that impact on the organisation |  |  |  |  |
| TOTAL STRATEGIC COMPLEXITY SCORE | */7* |  |  | */7* |

|  | *Agree* | | *Disagree* | | *Not applicable*  *or don’t know* | | *Likely to get more complex in next phase* | |
| --- | --- | --- | --- | --- | --- | --- | --- | --- |
| *TECHNICAL COMPLEXITIES* | | | | | | | |  |
| 1. The technology does not yet exist in a robust and dependable form | |  | |  | |  |  |  |
| 1. The technology is unfamiliar to the project team | |  | |  | |  |  |  |
| 1. The technology supply chain is not yet in place | |  | |  | |  |  |  |
| 1. The technology cannot be installed until the system is upgraded (e.g. hardware, bandwidth) | |  | |  | |  |  |  |
| 1. A key technology needs to be installed across multiple technical systems to achieve ‘integration’ | |  | |  | |  |  |  |
| 1. Introducing the technology will require significant changes in care pathways and organisational routines | |  | |  | |  |  |  |
| 1. Quality standards and regulatory requirements for using the technology in a health/care setting have not been fully defined (or key stakeholders don’t know about them or accept them) | |  | |  | |  |  |  |
| TOTAL TECHNICAL COMPLEXITY SCORE | | */7* | |  | |  | */7* |  |

|  | *Agree* | | *Disagree* | | *Not applicable*  *or don’t know* | | *Likely to get more complex in next phase* | |
| --- | --- | --- | --- | --- | --- | --- | --- | --- |
| *OPERATIONAL COMPLEXITIES* | | | | | | | | |
| 1. A schedule and resource plan have not yet been defined | |  | |  | |  | |  |
| 1. The pace of the project (time to achieve key goals and milestones) seems unachievable | |  | |  | |  | |  |
| 1. The budget is insufficient for the task or there is limited flexibility in how the budget can be used | |  | |  | |  | |  |
| 1. Resources (e.g. test facilities, equipment) may not be available when needed | |  | |  | |  | |  |
| 1. Evaluation measures and metrics have not yet been agreed | |  | |  | |  | |  |
| 1. Accurate, timely and comprehensive data reporting will be difficult or impossible | |  | |  | |  | |  |
| 1. New management tools and data sources will be needed to guide, monitor and evaluate the project | |  | |  | |  | |  |
| TOTAL OPERATIONAL COMPLEXITY SCORE | | */7* | |  | |  | | */7* |

|  | *Agree* | | *Disagree* | | *Not applicable*  *or don’t know* | | *Likely to get more complex in next phase* | |
| --- | --- | --- | --- | --- | --- | --- | --- | --- |
| *PEOPLE-RELATED COMPLEXITIES* | | | | | | | | |
| 1. The people leading the project are inexperienced in this kind of work | |  | |  | |  | |  |
| 1. The people leading the project do not have adequate control over project staff (e.g. no direct reporting) | |  | |  | |  | |  |
| 1. There are not yet sufficient people with the right skills available to participate in the project. | |  | |  | |  | |  |
| 1. There are no key people who are wholly allocated to the work for the project | |  | |  | |  | |  |
| 1. Lines of responsibility for tasks and deliverables are not yet defined | |  | |  | |  | |  |
| 1. Team members have limited confidence in the technology or do not fully understand how to use it | |  | |  | |  | |  |
| 1. Team members have limited motivation and are not yet functioning well as a team | |  | |  | |  | |  |
| TOTAL PEOPLE-RELATED COMPLEXITIES | | */7* | |  | |  | | */7* |

|  | *Agree* | | *Disagree* | | *Not applicable*  *or don’t know* | | *Likely to get more complex in next phase* | |
| --- | --- | --- | --- | --- | --- | --- | --- | --- |
| *“POLITICAL” COMPLEXITIES* | | | | | | | | |
| 1. The work does not have a senior sponsor in the organisation who recognises its importance and helps negotiate its progress | |  | |  | |  | |  |
| 1. The senior management team in the relevant department does not fully support the work | |  | |  | |  | |  |
| 1. Substantial work will be needed to bring people on board and develop a shared vision for the change | |  | |  | |  | |  |
| 1. People beyond the core team don’t understand the project or have unrealistic expectations for it | |  | |  | |  | |  |
| 1. People beyond the project team don’t support the project or are not aligned or have insufficient time | |  | |  | |  | |  |
| 1. The core team does not have the authority to make decisions | |  | |  | |  | |  |
| 1. The work will require cooperation across sectors (e.g. health / social care) | |  | |  | |  | |  |
| TOTAL “POLITICAL” COMPLEXITY SCORE | | */7* | |  | |  | | */7* |

Plot your scores on the radar charts below to get a quick visualisation of the different complexities as assessed by you. The one on the left is your assessment of current complexity (orange columns above); on the right is your assessment of emergent complexity (blue columns above). Compare your radar charts with those made by your colleagues. Do your charts look the same? If not, where are the discrepancies and what explains these?

| **Assessment of current complexity** | **Assessment of emergent complexity (how things will unfold in the future)** |
| --- | --- |
