# Supplementary material for: The NASSS-CAT Tools for Understanding, Guiding, Monitoring, and Researching Technology Implementation Projects in Health and Social Care: Protocol for an Evaluation Study in Real-World Settings
Source: JMIR Res Protoc. 2020 May 13;9(5):e16861. doi: 10.2196/16861 (PMC7254278; doi:10.2196/16861)
Supplement: Multimedia Appendix 5 [file resprot_v9i5e16861_app5.docx]

**NASSS-CAT (INTERVIEW VERSION)**

**RESEARCHING IMPLEMENTATION OF TECHNOLOGY PROJECTS**

© Professor Trish Greenhalgh, University of Oxford, and mHabitat

*Introduction*

This set of prompts for semi-structured interviews covers the different domains in the NASSS framework shown below. It will need to be adapted to suit the particular project that you are researching.

**
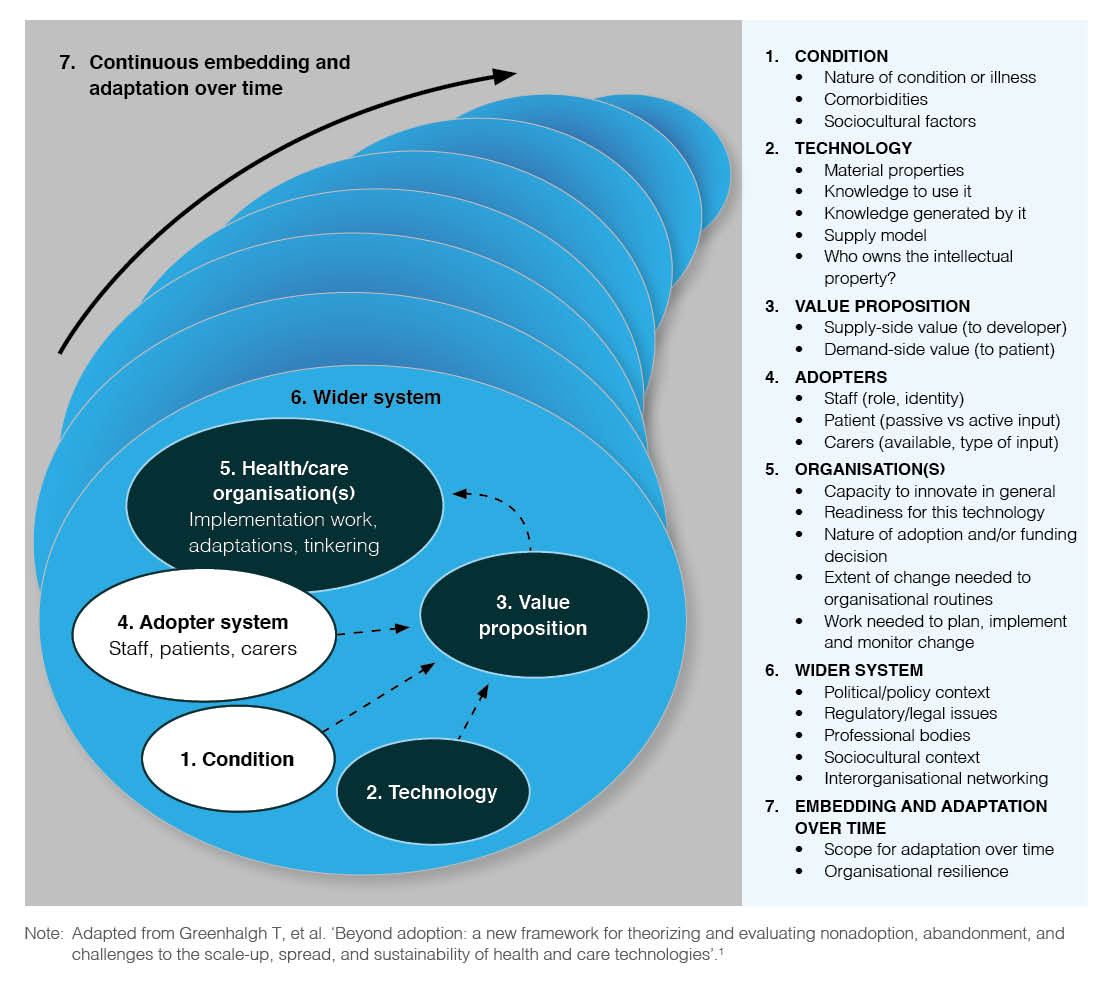
**

**Diagram: The NASSS framework** (© Greenhalgh at al J Med Internet Research 2017; 19 (11): e367)

## THE CONDITION OR ILLNESS

*Ask these questions of a clinician, social worker or someone else who understands the underlying illness or condition for which the technology was designed*

1. Tell me about the illness or condition for which this technology was [is being] designed.

*Prompts:*

- *Is the condition well-defined? How much is known about it?*
- *Do you know how many people are be affected by the condition, and in what way?*
- *Does the condition affect people in different ways? If so, can you give examples?*
- *Would people with the condition need input from more than one specialist or be in more than one care pathway?*

1. Do people with the condition tend to have other illnesses or factors that need to be taken into account when designing the technology?

*Prompts:*

- *Are there physical co-morbidities (e.g. does it affect the very old or those with a pre-existing condition)?*
- *Do some of the people affected have cognitive impairment, learning difficulties or communication difficulties?*
- *Do people with the condition tend to be in multiple care pathways or see more than one provider?*

1. Do the target population for this technology have social or cultural factors that need to be taken into account when designing the technology?

*Prompts:*

- *Are some of them likely to be socio-economically disadvantaged, homeless or socially excluded?*
- *Might some have religious restrictions or expectations that would affect how they manage their condition and their acceptance of technologies?*
- *Are some likely to have low health literacy (poor understanding of what is wrong and how to manage it)?*
- *Are some likely to have low system literacy (poor understanding of how to navigate the health or care system)?*
- *Are some likely to have low digital literacy (poor understanding of technologies and how to use them)?*
- *Are some of them likely to have problems understanding the language used by staff?*

1. How do you think the condition and the population it affects might change over the next 3-5 years?

*Prompts:*

- *Is the prevalence likely to increase or decrease?*

## THE TECHNOLOGY (or other innovation)

*Ask these questions of the technology developer or someone else who knows the design aspects and technological detail.*

1. What exactly is the technology?

*Prompts:*

- *Does it exist yet? If not, how much uncertainty is there around what it will be like?*
- *Is the technology easy to define – or are there some vague elements e.g. connects with hidden infrastructure, supplier does not disclose full details?*
- *Is the current version of the technology the definitive version?*

1. Where will the technology come from?

*Prompts:*

- *Has a supplier been identified?*
- *Is the supply chain in place?*
- *Is the technology easily substitutable (e.g. if the supplier withdrew, would it be possible to get this or a similar technology from elsewhere)?*

1. How would you rate the technology’s performance and dependability?

*Prompts:*

- *Does it capture, and where appropriate transmit, data accurately and reliably?*
- *Are there any privacy or security concerns?*

1. To what extent do you think the technology is usable by, and acceptable to, its intended users?

*Prompts:*

- *Can people try it out before committing to it?*
- *Do they understand what it does and the data it generates?*
- *What are the challenges of actually using it in practice?*
- *Have you observed people trying to use it? What do they say?*
- *What help do you offer users (e.g. helpdesk, hands-on support)? What has been the experience of supporting the introduction of the technology?*

1. Are there any key technical interdependencies?

*Prompts:*

- *Are there plans to make the technology connect with existing infrastructure?*
- *Does it need to be installed across multiple technical systems to achieve ‘integration’?*
- *Will there need to be an upgrade to the organisation’s IT system (e.g. new hardware, better bandwidth) to support use of the technology across the organisation?*
- *Would any target users have to upgrade their personal device(s) or home IT system?*

1. To what extent do you think this technology implies major changes to the way healthcare is delivered?

*Prompts:*

- *To what extent would implementing the technology require staff to do their jobs in a different way and/or interact with different people or teams?*
- *To what extent would implementing the technology require new or different steps in the care pathway (e.g. new administrative processes)?*

1. To what extent do you think the technology (and/or the service model it supports) will become obsolete or require updating in the next 3-5 years?

*Prompts:*

- *To what extent can the technology be adapted to take account of future changes?*
- *To what extent will the technology supply model change?*

## THE VALUE PROPOSITION (costs and benefits of the technology)

*Ask these questions of the technology developer or business lead for the organisation.*

1. What is the commercial value of the technology?

*Prompts:*

- *If the technology does not yet exist in a definitive form, how strong is the case for investing in its [further] technical development?*
- *Is there a plausible business case for developing the technology (including up-front investment, a well-defined customer base and market drivers, consideration of competing products and realistic assessment of challenges of implementing at scale in a public-sector health or care environment)?*

1. What is the value of the technology to the patient or client?

*Prompts:*

- *Are there any high-quality studies (e.g. randomised controlled trials) to demonstrate the technology’s efficacy for this patient/client group?*
- *What evidence is there that the technology’s benefits outweigh its potential harms?*
- *Have the technology’s efficacy and safety been measured in terms of an outcome that matters to patients/clients?*

1. What is the value of the technology to the clinician or other professional?

*Prompts:*

- *Does the technology create work – if so, for whom?*
- *Have the technology’s benefits to the clinician been shown to outweigh the hassle of using it?*

1. What is known about the value that this technology could bring to the health or care system?

*Prompts:*

- *Has the technology (or the technology-supported care model) been shown to have an overall advantage over existing practice?*
- *Has technology been shown to be effective and cost-effective in terms of how much benefit it will bring for a given financial outlay?*
- *Are there any safety concerns about the technology or the care model it supports?*
- *Has this technology-supported care model been successfully implemented in a similar context to the one being contemplated?*
- *Are there concerns that the technology, whilst improving care for some patients, could widen inequalities?*
- *Are regulatory and other approvals for the technology in place?*

1. What is known about the value that this technology is likely to being to this particular organisation?

*Prompts:*

- *Will new technical infrastructure be needed?*
- *Will the organisation need to introduce substantial changes to organisational routines and pathways?*

1. Could the technology generate a negative value (i.e. costs would be more than gains) for some stakeholders?

*Prompts:*

- *Potential loss of income?*
- *Destabilising a provider?*
- *Hidden or knock-on costs?*

1. Is the value proposition likely to change over the next 3-5 years?

*Prompts:*

- *A new, better technology is on the horizon?*
- *The market for the technology could change significantly?*
- *A key regulatory decision could be made or reversed?*

## THE INTENDED ADOPTERS OF THE TECHNOLOGY

*Ask these questions of anyone who uses, or is expected to use, the technology.*

1. What do patients and carers think of the technology?

*Prompts:*

- *Does the technology require substantial input from the patient or their immediate carer?*
- *What is the meaning of the technology to the patient? (Do they like using it? Do they mind having it in their home? Does it remind them of an illness they would rather forget about?)*
- *What are the practicalities of using the technology? (Are they prepared to learn to use it? Can they make it work? If not, why not?)*

1. What do front-line staff think of the technology?

*Prompts:*

- *Do staff question the value proposition for the technology (e.g. do they feel that adopting it would jeopardise the quality or safety of patient care, or do they think it is more time-consuming than existing practice)?*
- *Would the technology require staff to do their jobs differently and perhaps take on a new, unwanted, role and identity (e.g. 'data entry person')?*
- *Do individuals or teams have the resources, time, space or support to learn to use the technology?*
- *Are staff confident to be creative and flexible when implementing technologies, and is there organisational support for this adaptive approach?*

1. Are there people who are indirectly affected by the technology?

*Prompts:*

- *Would technology require input from others (e.g. relatives, care home staff), who may be unable or unwilling to learn to use it?*
- *Would the technology would make someone else’s job obsolete or more difficult?*

1. How do you anticipate that individual users’ perceptions of the technology will change over the next 3-5 years?

*Prompts:*

- *Do you think patients or their lay carers are likely to change their views on the technology?*
- *Do you think key staff groups are likely to change their views on the technology?*

## THE ORGANISATION(S)

*Ask these questions of people who know the organisation and the challenges it faces e.g. board member, human resources lead, staff representative.*

1. How would you rate the organisation’s overall capacity to take on technological innovations?

*Prompts:*

- *How strong is the leadership?*
- *Are the organisation’s mission and values are clear?*
- *How good are internal relations, especially between managers and clinicians?*
- *Would you describe the management structure as flat and egalitarian or top-down and hierarchical? (For example, are individual departments discouraged from horizon-scanning for new products and ideas, and are they frowned upon if they introduce innovations?)*
- *What is the organisation’s track record of introducing any kind of change?*
- *To what extent are there slack resources (people or money) to channel into innovative projects?*
- *To what extent is it a learning organisation (in which staff are encouraged to meet and talk about new ideas and projects, there are measures in place to capture data and monitor progress, and risk-taking is encouraged?*
- *What is the current level of digital maturity?*

1. To what extent do you think the organisation is ready for this particular technology/innovation?

*Prompts:*

- *Is there a good fit between the organisation's mission and the innovation?*
- *Are there any key people (especially senior management) who oppose the innovation or are unconvinced of its value?*
- *Is the business case strong and accepted?*
- *Are the implications (e.g. work required) of introducing, implementing and evaluating the technology have been adequately and realistically assessed?*
- *If money is needed, has a budget line been allocated?*

1. To what extent do you think organisational routines, pathways and processes will need to change to accommodate the technology/innovation?

*Prompts:*

- *Will different kinds of staff (e.g. new hires) need to be involved in the process or pathway once the technology has been introduced?*
- *Will a new (or radically revised) process or pathway will need to be developed?*
- *Will the core process or pathway need to link differently with other key processes and pathways in the organisation?*

1. Are there any relevant challenges in procurement processes that would make it harder for the organisation to invest in this technology?

*Prompts: For example…*

- *Is the provider on the organisation’s procurement framework*
- *Are there any existing contracts that need to expire first?*
- *Are there aspects of the procurement process that are not yet clear (e.g. Who will fund this? Who will be liable for costs? Is there an identified budget? It is capital or revenue? Is the funding recurrent? Are there issues with timing/accruals of funding?)*

1. To what extent do you think the work of implementation has been realistically assessed and adequately resourced?

*Prompts:*

- *Work to bring people on board and develop a shared, organisation-wide vision for the change?*
- *Work to develop, implement and mainstream new care pathways and processes?*
- *Work to coordinate the project across more than one organisation or sector?*
- *Work to evaluate and monitor the change?*

1. To what extent do you think the organisation(s) are likely to have significant restructurings or changes in leadership, mission or strategy over the next 3-5 years in a way that will impact on this project?

## THE EXTERNAL CONTEXT FOR INNOVATION

*Ask these questions of people work outside the organisation or a ‘horizon-scanner’ who looks beyond the organisation.*

1. What do you think of the political/policy climate as it relates to this innovation?

*Prompts:*

- *External political or economic changes impacting on the organisation which could threaten the introduction of the innovation?*
- *Current policy priorities that conflict with this initiative?*

1. Do you think professional organisations support or oppose this innovation?

*Prompts:*

- *Concerns about quality or safety of care?*
- *Concerns about confidentiality and wider information governance?*
- *Concerns about professional workload?*
- *Other pressing priorities?*

1. Do you think patient organisations and lobbying groups are likely to support or oppose this innovation?

*Prompts:*

- *Concerns about quality or safety of care?*
- *Concerns about privacy and/or what will happen to the data*
- *Other pressing priorities?*

1. Is the regulatory context supportive or adverse for this innovation?

*Prompts:*

- *Have quality standards and regulatory requirements for using the technology in a health or care setting been fully defined?*
- *Do key stakeholders do not know about and accept these standards and requirements?*

1. Is the commercial context supportive or adverse for this innovation?

*Prompts:*

- *Does the technology industry view this innovation (or similar products) positively or negatively?*
- *Does the technology use industry-standard components?*

1. Are there opportunities for learning from other organisations?

*Prompts:*

- *Is the innovation up and running elsewhere – or are others planning to implement it in a similar time frame?*
- *Are there established inter-organisational knowledge exchange networks (e.g. quality improvement collaboratives) or could these be set up?*

1. Are there other external changes that could threaten introduction of the technology?
2. How do you think the policy, regulatory and economic context for this innovation is likely to change over the next 3-5 years? Is there likely to be turbulence?

*Prompts:*

- *Change of government?*
- *New policy priorities?*
- *Economic recession?*
- *New regulatory framework?*
- *Withdrawal of industry commitment?*
